# Claudin-4-adhesion signaling drives breast cancer metabolism and progression via liver X receptor β

**DOI:** 10.1186/s13058-023-01646-z

**Published:** 2023-04-14

**Authors:** Yuko Murakami-Nishimagi, Kotaro Sugimoto, Makoto Kobayashi, Kazunoshin Tachibana, Manabu Kojima, Maiko Okano, Yuko Hashimoto, Shigehira Saji, Tohru Ohtake, Hideki Chiba

**Affiliations:** 1 Department of Basic Pathology, Fukushima Medical University School of Medicine, Fukushima, 960-1295 Japan; 2 Department of Breast Surgery, Fukushima Medical University School of Medicine, Fukushima, 960-1295 Japan; 3 Department of Obstetrics and Gynecology, Fukushima Medical University School of Medicine, Fukushima, 960-1295 Japan; 4 Department of Diagnostic Pathology, Fukushima Medical University School of Medicine, Fukushima, 960-1295 Japan; 5 Department of Medical Oncology, Fukushima Medical University School of Medicine, Fukushima, 960-1295 Japan

**Keywords:** Tight junction, Claudin, Cell adhesion signal, Nuclear receptor, Triple-negative breast cancer

## Abstract

**Background:**

Cell adhesion is indispensable for appropriate tissue architecture and function in multicellular organisms. Besides maintaining tissue integrity, cell adhesion molecules, including tight-junction proteins claudins (CLDNs), exhibit the signaling abilities to control a variety of physiological and pathological processes. However, it is still fragmentary how cell adhesion signaling accesses the nucleus and regulates gene expression.

**Methods:**

By generating a number of knockout and rescued human breast cell lines and comparing their phenotypes, we determined whether and how CLDN4 affected breast cancer progression in vitro and in vivo. We also identified by RNA sequencing downstream genes whose expression was altered by CLDN4-adhesion signaling. Additionally, we analyzed by RT-qPCR the CLDN4-regulating genes by using a series of knockout and add-back cell lines. Moreover, by immunohistochemistry and semi-quantification, we verified the clinicopathological significance of CLDN4 and the nuclear receptor LXRβ (liver X receptor β) expression in breast cancer tissues from 187 patients.

**Results:**

We uncovered that the CLDN4-adhesion signaling accelerated breast cancer metabolism and progression via LXRβ. The second extracellular domain and the carboxy-terminal Y197 of CLDN4 were required to activate Src-family kinases (SFKs) and the downstream AKT in breast cancer cells to promote their proliferation. Knockout and rescue experiments revealed that the CLDN4 signaling targets the AKT phosphorylation site S432 in LXRβ, leading to enhanced cell proliferation, migration, and tumor growth, as well as cholesterol homeostasis and fatty acid metabolism, in breast cancer cells. In addition, RT-qPCR analysis showed the CLDN4-regulated genes are classified into at least six groups according to distinct LXRβ- and LXRβS432-dependence. Furthermore, among triple-negative breast cancer subjects, the "CLDN4-high/LXRβ-high" and "CLDN4-low and/or LXRβ-low" groups appeared to exhibit poor outcomes and relatively favorable prognoses, respectively.

**Conclusions:**

The identification of this machinery highlights a link between cell adhesion and transcription factor signalings to promote metabolic and progressive processes of malignant tumors and possibly to coordinate diverse physiological and pathological events.

**Supplementary Information:**

The online version contains supplementary material available at 10.1186/s13058-023-01646-z.

## Background

Breast cancer represents the most common malignancy in women, with an increased incidence [1, 2]. In 2020, 2.3 million people were estimated to be diagnosed with breast cancer worldwide, and 685,000 people died of this tumor [3]. Breast cancer is classified by the gene expression profiles into intrinsic subtypes, such as luminal A, luminal B, HER2 (human epidermal growth factor receptor 2), and basal-like, which possess distinct biological properties, drug responses, and patient outcomes [1, 4–6]. More practically, the prognosis predictions and therapeutic strategies are immunohistochemically determined by the estrogen receptor (ER), progesterone receptor (PgR), and HER2 status, as well as by Ki-67/MIB-1 proliferation index [7, 8]. Patients positive for either ER, PgR, or HER2 exhibit a relatively favorable prognosis owing to valid medication against these receptors. By contrast, triple-negative breast cancer (TNBC), which accounts for 15–20% of breast cancer, shows limited therapeutic options and poor outcomes [9, 10].

Cell adhesion molecules, such as E-cadherin, are necessary for multicellular organisms to maintain tissue architecture and homeostasis. They were considered to function as tumor suppressor proteins, but it is an oversimplified principle [11]. For instance, E-cadherin contributes to collective invasion and/or metastasis in the breast cancer [12, 13]. In addition to the adhesive activity, cell adhesion proteins exhibit signaling properties that coordinate a wide range of cell behaviors [14–18], theoretically via activation or repression of transcription factors that control the expression of target genes [19]. However, it remains poorly established how cell adhesion signaling reaches the nucleus and regulates gene expression.

The claudin (CLDN) family is the structural and functional backbone of tight junctions and contains a short cytoplasmic N-terminus, two extracellular loops (EC1 and EC2), and a C-terminal cytoplasmic domain [20–25]. We have recently identified that CLDN6-adhesion signaling regulates the nuclear receptor activity [20–23]. In brief, we uncovered that CLDN6 couples with Src-family kinases (SFKs) in the EC2-dependent and the C-terminal Y196/200-dependent manners. We also showed that the CLDN6/SFK/PI3K/AKT axis targets the AKT phosphorylation sites in the retinoic acid receptor γ (RARγ) and estrogen receptor α (ERα) and stimulates their activities independently of ligands. Importantly, these phosphorylation motifs (RXXS, aa 515 to 518 in human ERα) are conserved in 14 of 48 members of human nuclear receptors, further suggesting the biological relevance of these phosphorylation sites.

CLDNs also possess aberrant expression and/or subcellular localization in a broad range of cancer types [26–28], leading to the promotion or repression of tumor progression, possibly via the dysregulated CLDN signaling [19]. In breast cancer, the CLDN-low subtype, which is characterized by the low expression of cell–cell adhesion molecules such as CLDN3/4/7, had been considered to show immature cancer properties and poor prognosis [33–35]. However, it has been recently reported that the CLDN-low tumor displays heterogeneous phenotypes but neither an independent intrinsic subtype nor a poor outcome [33, 34]. On the other hand, there are conflicting reports on the relationship between high CLDN4 expression and patient prognosis in the breast cancer [35–37]. We have recently demonstrated that aberrant CLDN6 signaling accelerates endometrial cancer progression in vitro and in vivo by hijacking the CLDN6–ERα axis [38, 39]. Among the CLDN family, CLDN4 is evolutionarily close to CLDN6 [40]. Additionally, Y196 and Y200 in the C-terminal cytoplasmic domain of human/mouse CLDN6 are required to propagate intracellular signals [18, 39] and are conserved in human/mouse CLND4Y193/197 [19]. Taken together, we hypothesized that the CLDN4 signaling might regulate breast cancer progression by regulating the nuclear receptor activity in a similar mechanism to CLDN6 in endometrial cancer.

Here, we show that aberrant CLDN4 signaling advances breast cancer metabolism and progression via liver X receptor β (LXRβ), a member of the nuclear receptor family. We also demonstrate that the CLDN4 signaling activates SFK/AKT and targets LXRβS432, resulting in stimulation of the LXRβ activity and malignant behaviors in breast cancer cells. Moreover, we present that the "CLDN4-high/LXRβ-high" group in TNBC cases reveals significantly shorter overall and recurrence-free survival than the "CLDN4-low and/or LXRβ-low" group.

## Results

### CLDN4 drives breast cancer progression

We first determined the CLDN4 expression in four representative human breast cancer cell lines, MCF-7, T47D, SKBR-3, and MDA-MB-231. On Western blot analysis, CLDN4 protein was expressed in the ERα^+^/PgR^+^ cell lines MCF-7 and T47D, as well as in the ERα^–^/PgR^–^/HER2^+^ cell line SKBR-3, but hardly detected in the TNBC cell line MDA-MB-231 (Additional file 1: Fig. S1A). Immunofluorescence staining revealed that CLDN4 was observed along the cell borders in MCF-7, T47D, and SKBR-3 cells but not in MDA-MB-231 cells (Additional file 1: Fig. S1B).

**Fig. 1:**
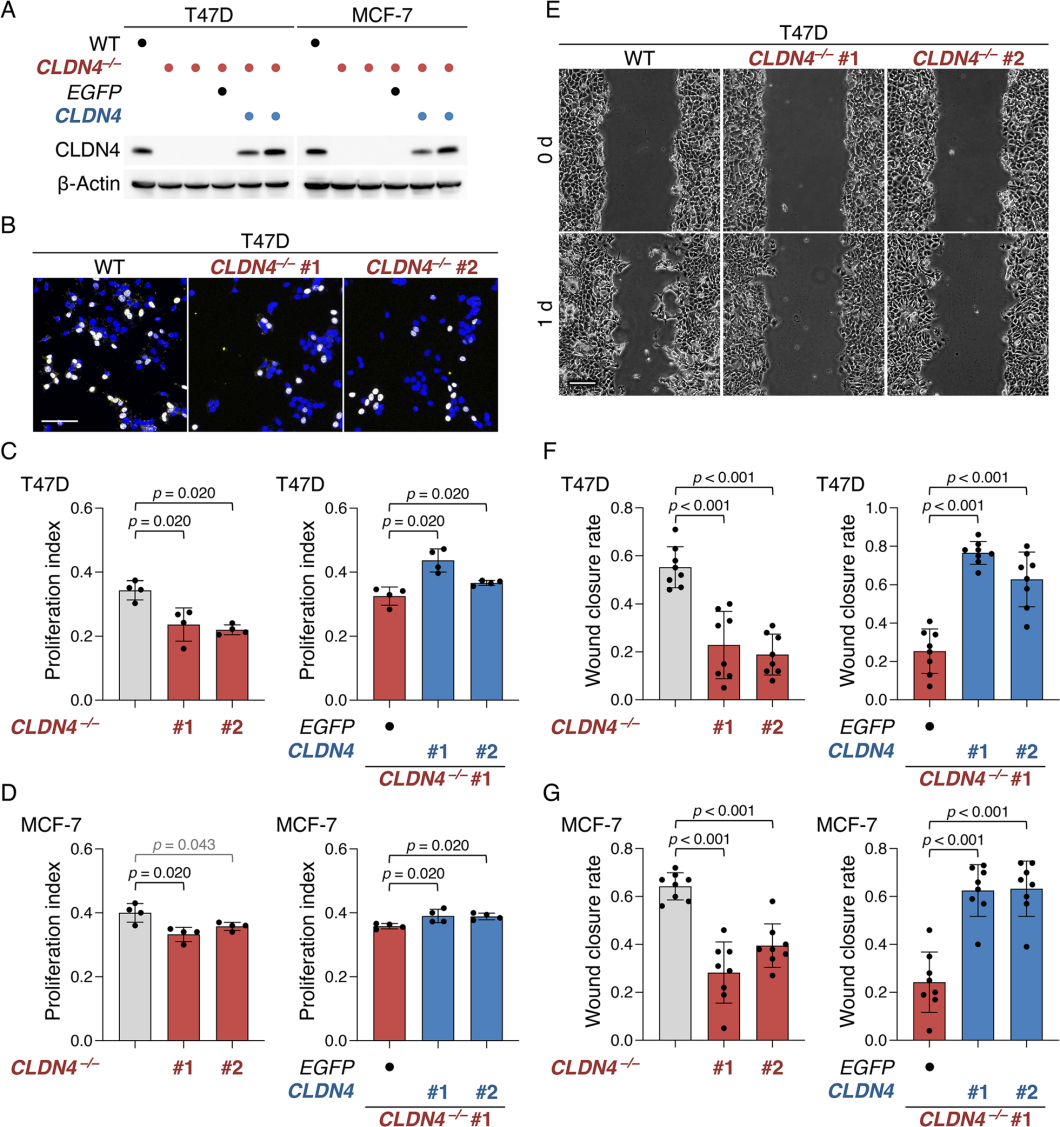
CLDN4 promotes malignant behavior of the breast cancer cell lines T47D and MCF-7. **A** Western blot analysis showing the absence and presence of CLDN4 protein in the indicated cells. **B**–**D** Representative and quantitative BrdU assay for the indicated cells. The BrdU/DAPI levels are plotted and shown in the histograms (mean ± SD; n = 4). **E**–**G** Typical and quantitative wound healing assay of the indicated cells. The wound closure rates are plotted and shown in the histograms (mean ± SD; n = 8). Scale bars, 50 µm (**B**); 100 μm (**E**)

To evaluate the involvement of CLDN4 in breast cancer progression, we subsequently established both T47D:*CLDN4*^*–/–*^ and MCF-7:*CLDN4*^*–/–*^ cell lines using CRISPR/Cas9-based genome editing. We also generated T47D:*CLDN4*^*–/–*^:*CLDN4* and MCF-7:*CLDN4*^*–/–*^:*CLDN4* cells, and compared the phenotypes in these knockout (KO) and rescued cell lines with those in the respective parental cells. KO of *CLDN4* gene in both T47D and MCF-7 cells was verified by DNA sequencing (Additional file 1: Fig. S2A), and the loss or re-expression of CLDN4 protein in these cells was confirmed by Western blot (Fig. 1A). The absence and presence of CLDN4 in T47D and MCF-7 cells did not influence their morphological appearance (Additional file 1: Fig. S3A, B). BrdU assay revealed that cellular proliferation was significantly decreased in two clones of T47D:*CLDN4*^*–/–*^ and MCF-7:*CLDN4*^*–/–*^ cells compared with T47D and MCF-7 cells, respectively (Fig. 1B–D). In addition, the re-expression of CLDN4 in T47D:*CLDN4*^*–/–*^ and MCF-7:*CLDN4*^*–/–*^ cells led to a significant increase in cellular proliferation (Fig. 1C, D, right panels). The wound-healing assay demonstrated that cell migration in both clones of T47D:*CLDN4*^*–/–*^ and MCF-7:*CLDN4*^*–/–*^ cells was significantly reduced compared with that in the parental cells (Fig. 1E–G). Similar to cell proliferation, cell migration was rescued by the re-expression of CLDN4 in T47D:*CLDN4*^*–/–*^ and MCF-7:*CLDN4*^*–/–*^ cells (Fig. 1F, G, right panels). Furthermore, cell invasion was significantly diminished in T47D:*CLDN4*^*–/–*^ cells compared with T47D cells (Additional file 1: Fig. S4A, B). On the apoptosis assay, there were no significant differences in apoptosis between T47D and T47D:*CLDN4*^*–/–*^ cells (Additional file 1: Fig. S4C).

We next generated, using a lentiviral vector system, MDA-MB-231 cells expressing CLDN4 (MDA-MB-231:*CLDN4*; Additional file 1: Fig. S5A). As expected, cell proliferation, migration, and invasion were significantly increased in MDA-MB-231:*CLDN4* compared to those in MDA-MB-231 cells (Additional file 1: Fig. S5B–D). Thus, these results using three distinct cell lines indicated that CLDN4 accelerates malignant behaviors of breast cancer cells in vitro.

**Fig. 2:**
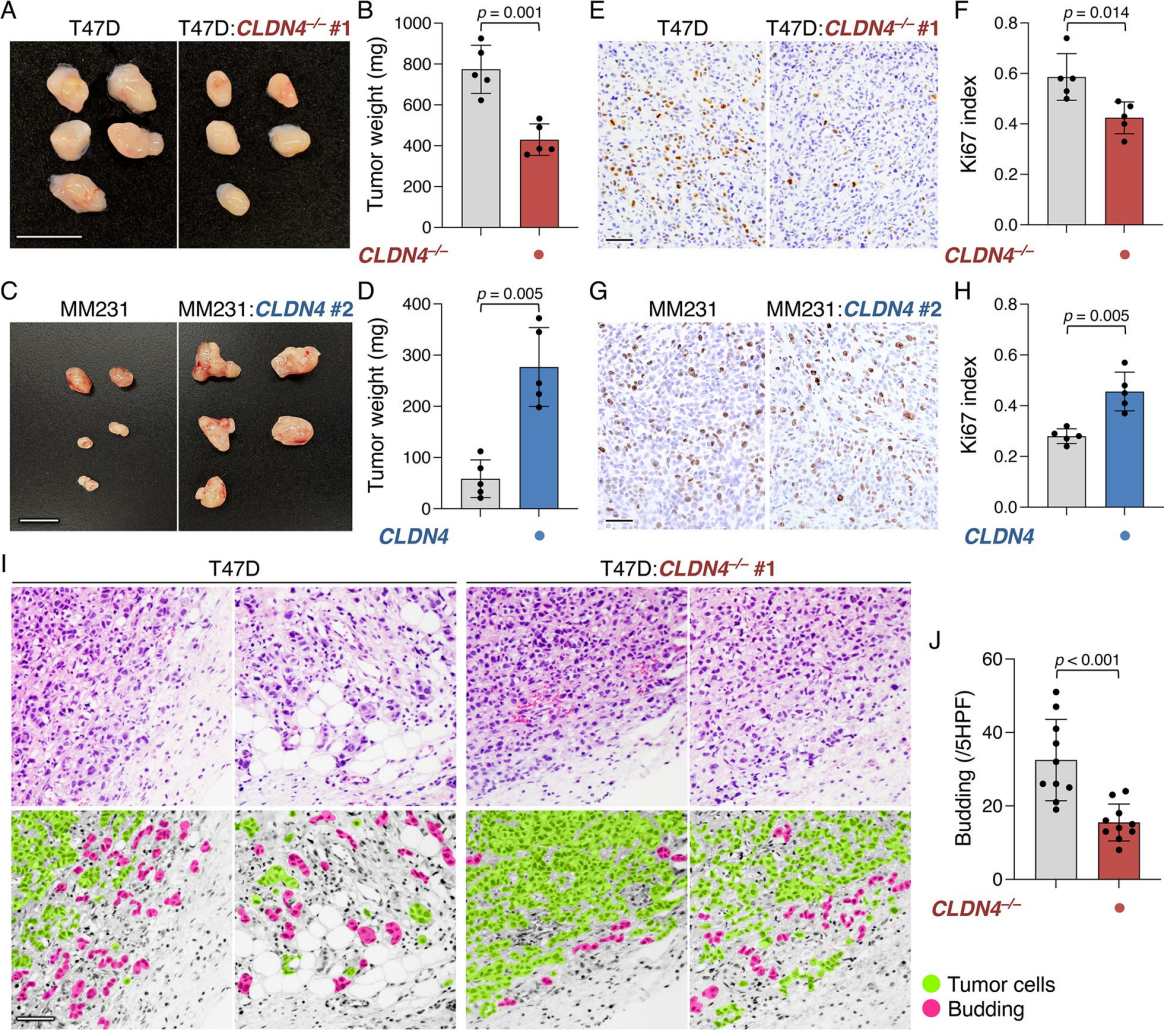
CLDN4 advances the malignant behavior of breast cancer cells in vivo. **A**–**D** Gross appearance and weight of the indicated xenografts at 28 days after the inoculation. T47D:*CLDN4*^*–/–*^ clone #1 and MDA-MB-231:*CLDN4* batch #2 were used for xenograft experiments. The tumor weight is plotted and shown in histograms (mean ± SD; n = 5). Similar results were obtained from xenograft experiments using different T47D:*CLDN4*^*–/–*^ clones and MDA-MB-231:*CLDN4* batches. **E**–**H** Typical images and quantification of Ki-67 labeling assay of the indicated xenografts. Ki-67 index is plotted and shown in the histograms (mean ± SD; n = 5). **I** Microscopic appearance of T47D and T47D:*CLDN4*^*–/–*^ xenografts. The regions corresponding to the xenograft tumors and the surrounding fibrous capsule are indicated. **J** Quantification of budding in T47D and T47D:*CLDN4*^*–/–*^ xenografts. The budding number is plotted and shown in the histograms (mean ± SD; n = 10). MM231, MDA-MB-231; HPF, high-power field. Scale bars, 1 cm (**A**, **C**); 100 μm (**E**, **G**, **I**)

We then verified whether CLDN4 also promoted breast cancer progression in vivo. Four weeks after inoculation in SCID (severe combined immunodeficiency) mice, the tumor growth of T47D:*CLDN4*^*–/–*^ and MDA-MB-231:*CLDN4* xenografts was decreased and increased compared with that of their parental cell xenografts, respectively (Fig. 2A–D). Neither lymph node nor distant metastasis was grossly evident in these xenografts. Microscopically, cellular proliferation was significantly reduced and enhanced in T47D:*CLDN4*^*–/–*^ and MDA-MB-231:*CLDN4* xenografts compared with that in T47D and MDA-MB-231 ones, respectively (Fig. 2E–H). Of note, invasion around the tumor, namely the budding of cancer cells, was significantly hampered in T47D:*CLDN4*^*–/–*^ xenografts compared with T47D xenografts (Fig. 2I, J). Because two clones of each T47D:*CLDN4*^*–/–*^ and MCF-7:*CLDN4*^*–/–*^ exhibited principally the same phenotypes, we used clone #1 among these cell lines for further analyses.

### The EC2 and Y197 of CLDN4 are required to activate SFKs in breast cancer cells

**Fig. 3:**
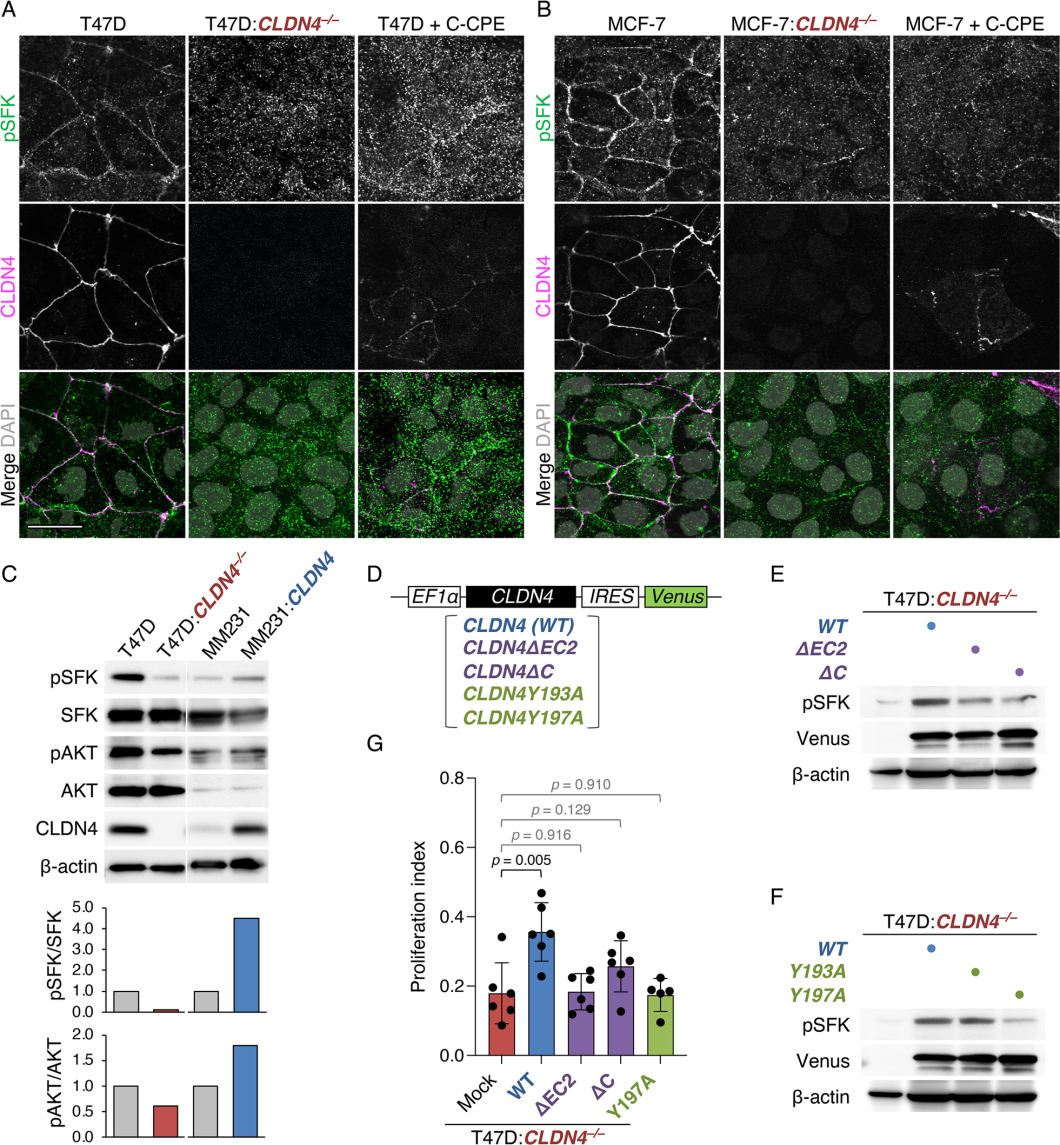
CLDN4 activates SFKs in breast cancer cells via the EC2 and Y197. **A**, **B** Confocal images of the indicated proteins in T47D:C*LDN4*^*–/–*^, MCF-7:*CLDN4*^*–/–*^ and their parental cells. T47D and MCF-7 cells were grown for 24 h in the presence or absence of 1.0 μg/ml C-CPE. Scale bar, 20 μm. **C** Western blot for the indicated proteins in the revealed breast cancer cells. The protein levels are normalized to the rehybridized β-actin levels, and the relative levels are shown in the histograms. MM231, MDA-MB-231. **D** The construct of wild-type (WT) and mutant CLDN4 expression vectors. EF-1α, elongation factor-1α; IRES, internal ribosome entry site. **E**, **F** Western blot for the indicated proteins in the revealed T47D cells. **G** Quantitative BrdU assay for the indicated cells. The BrdU/DAPI levels are plotted and shown in the histograms (mean ± SD; n = 5–6)

Since CLDN6 and CLDN11 are known to couple with SFKs [42, 43], we subsequently tested whether CLDN4 also activates SFKs. Double immunofluorescence staining showed that pSFK concentrated along cell boundaries with CLDN4 in T47D and MCF-7 cells, but hardly in T47D:*CLDN4*^*–/–*^ or MCF-7:*CLDN4*^*–/–*^ cells (Fig. 3A, B). On Western blot, the pSFK and the downstream pAKT levels were reduced and increased in T47D:*CLDN4*^*–/–*^ and MDA-MB-231:*CLDN4* cells compared with those in their parental cells, respectively (Fig. 3C).

We then validated the involvement of CLDN4-EC2, CLDN4-C, and CLDN4-Y193/197 in SFK activation using T47D:*CLDN4*^*–/–*^ cells expressing wild-type (WT) CLDN4 and the corresponding CLDN4 mutants (Fig. 3D). As shown in Fig. 3E, the pSFK levels in T47D:*CLDN4*^*–/–*^*:WT-CLDN4* cells were markedly induced compared with those in T47D:*CLDN4*^*–/–*^ cells, and they were higher than those in T47D:*CLDN4*^*–/–*^*:CLDN4ΔEC2* and T47D:*CLDN4*^*–/–*^*:CLDN4ΔC* cells. In addition, the induced pSFK levels in T47D:*CLDN4*^*–/–*^*:CLDN4Y193A* cells were similar to those in T47D:*CLDN4*^*–/–*^*:WT-CLDN4* cells, whereas those in T47D:*CLDN4*^*–/–*^*:CLDN4Y197A* cells were lower than those in T47D:*CLDN4*^*–/–*^*:WT-CLDN4* cells (Fig. 3F). Furthermore, the CLDN4-enhanced cell proliferation was reversed in T47D:*CLDN4*^*–/–*^*:CLDN4ΔEC2*, T47D:*CLDN4*^*–/–*^*:CLDN4ΔC,* and T47D:*CLDN4*^*–/–*^*:CLDN4Y197A* cells (Fig. 3G). When T47D and MCF-7 cells were exposed to the C-terminal half of *Clostridium Perfringens* enterotoxin (C-CPE), which binds to the EC2 of CLDNs and excludes them from cell membranes without changes in their total protein levels [18, 39, 42], the pSFK-immunoreactive signals were markedly reduced (Fig. 3A). Moreover, the SFK inhibitor PP2 and the AKT inhibitor VIII, as well as C-CPE, significantly inhibited cell proliferation in T47D cells (Additional file 1: Fig. S6). In contrast, the two inhibitors and C-CPE did not affect the cell growth of T47D cells in the absence of CLDN4. The CLDN4-triggered cell proliferation was completely prevented in MDA-MB-231:*CLDN4ΔC* cells, further showing the importance of CLDN4-C in breast cancer progression (Additional file 1: Fig. S5B). Hence, these results indicated that the CLDN4 signaling activates SFK/AKT and accelerates breast cancer progression in the EC2- and the C-terminal Y197-dependent manners.

### The CLDN4 signaling modulates the expression of genes related to cholesterol homeostasis and fatty acid metabolism in breast cancer cells

**Fig. 4:**
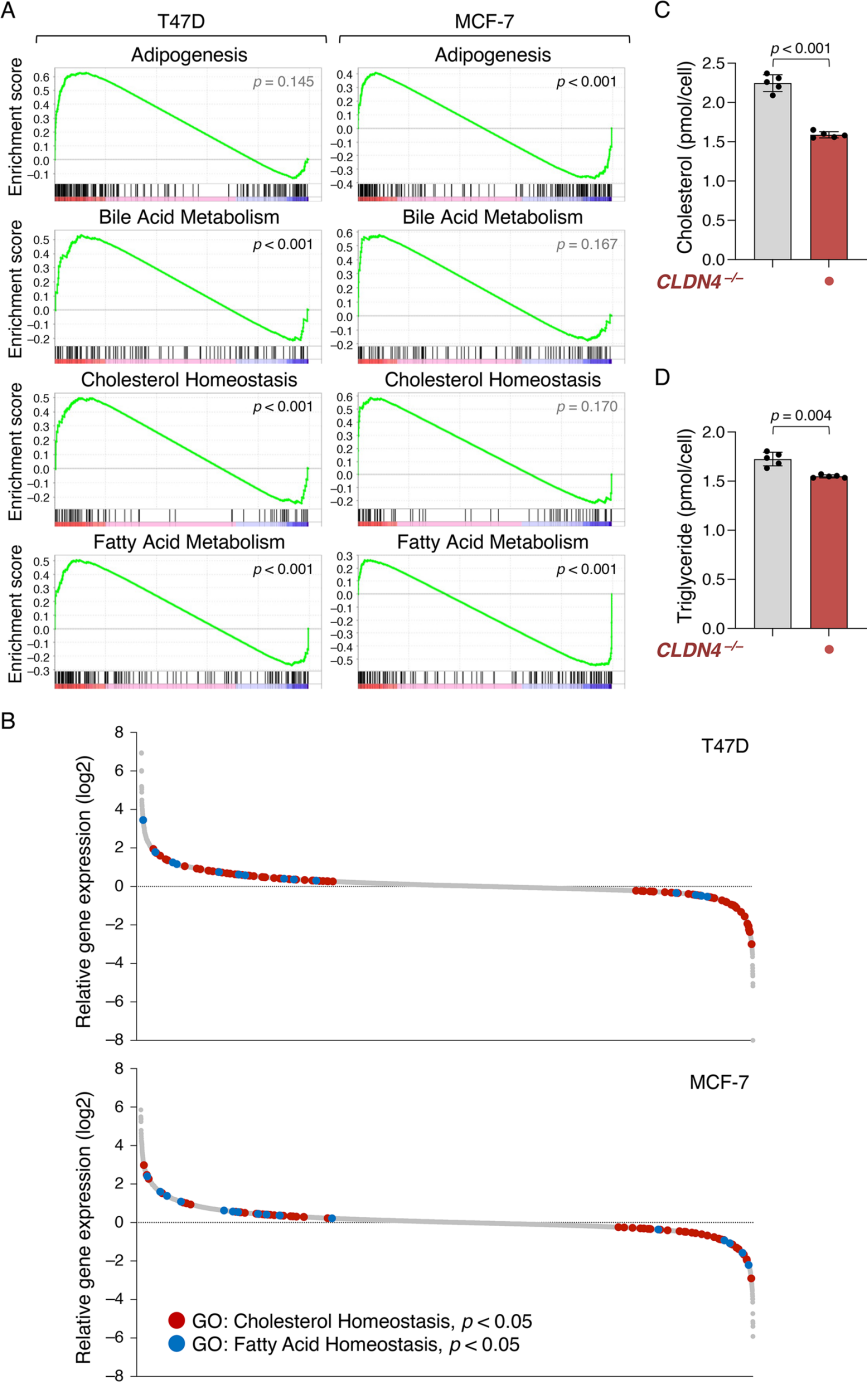
The CLDN4 signaling regulates the expression of genes relating to cholesterol homeostasis and fatty acid metabolism in breast cancer cells. **A** Gene set enrichment analysis (GSEA) of ranked differential gene scores showing the CLDN4-dependent enrichment of gene sets of adipogenesis, bile acid metabolism, cholesterol homeostasis, and fatty acid metabolism in T47D and MCF-7 cells. **B** Waterfall plot showing the alteration of the gene expression by CLDN4-KO in T47D and MCF-7 cells. Genes showing significant changes in gene ontology (GO) cholesterol homeostasis (GO:0042632) and fatty acid homeostasis (GO:0055089) are indicated in red and blue dots, respectively. **C**, **D** The intracellular levels of cholesterol (**C**) and triglyceride (**D**) in T47D and T47D:*CLDN4*^*–/–*^ cells are plotted and shown in histograms (mean ± SD; n = 5)

To identify downstream genes whose expression is altered by the CLDN4-adhesion signaling, we next compared, using RNA sequencing, the transcriptomes in T47D:*CLDN4*^*–/–*^ and MCF-7:*CLDN4*^*–/–*^ cells with those in T47D and MCF-7 cells, respectively (Fig. 4A, B; Additional file 1: Fig. S7). Gene set enrichment analysis (GSEA) of ranked differential gene scores revealed that gene sets of adipogenesis, bile acid metabolism, cholesterol homeostasis, and fatty acid metabolism were CLDN4-dependently and significantly enriched in T47D and/or MCF-7 cells (Fig. 4A). Additionally, gene ontology analysis disclosed that a variety of genes involved in cholesterol and fatty acid homeostasis were highly modulated in both T47D:*CLDN4*^*–/–*^ and MCF-7:*CLDN4*^*–/–*^ cell lines compared with their parental cells (Fig. 4B). As expected, intracellular levels of cholesterol and triglyceride in T47D:*CLDN4*^*–/–*^ cells were significantly decreased compared with those in T47D cells (Fig. 4C, D). Furthermore, among the CLDN4-activated genes, which expression was significantly decreased in both *CLDN4*^*–/–*^ cell lines, there were many gene products that are known to be associated with malignant phenotypes (Additional file 1: Fig. S7). Interestingly, several LXR target genes, such as *LDLRAD4* (Low-density lipoprotein receptor class A domain-containing protein 4), *DKK1* (Dickkopf WNT signaling pathway inhibitor 1), *KRT80* (Keratin 80), and *FBP1* (Fructose-bisphosphatase 1), were included. Taken collectively with the finding showing that LXRs greatly contribute to maintaining cholesterol homeostasis and fatty acid metabolism in normal and cancer cells [43, 44], these results suggested that CLDN4 promotes breast cancer metabolism through LXRs.

### The CLDN4 signaling targets LXRβ to accelerate breast cancer metabolism and progression

We subsequently verified the LXRα/β expression in human breast cancer cells. RT-qPCR analysis showed that *LXRβ* mRNA was expressed in T47D, MCF-7, and MDA-MB-231 cells, whereas *LXRα* transcripts were hardly detected in these cell lines (Additional file 1: Fig. S8A). On Western blot, LXRβ but not LXRα protein was detected in T47D, MCF-7, and MDA-MB-231 cells (Additional file 1: Fig. S8B). Immunofluorescent staining revealed that LXRβ but not LXRα appeared to be observed in the nuclei of these cell lines (Additional file 1: Fig. S8C).

**Fig. 5:**
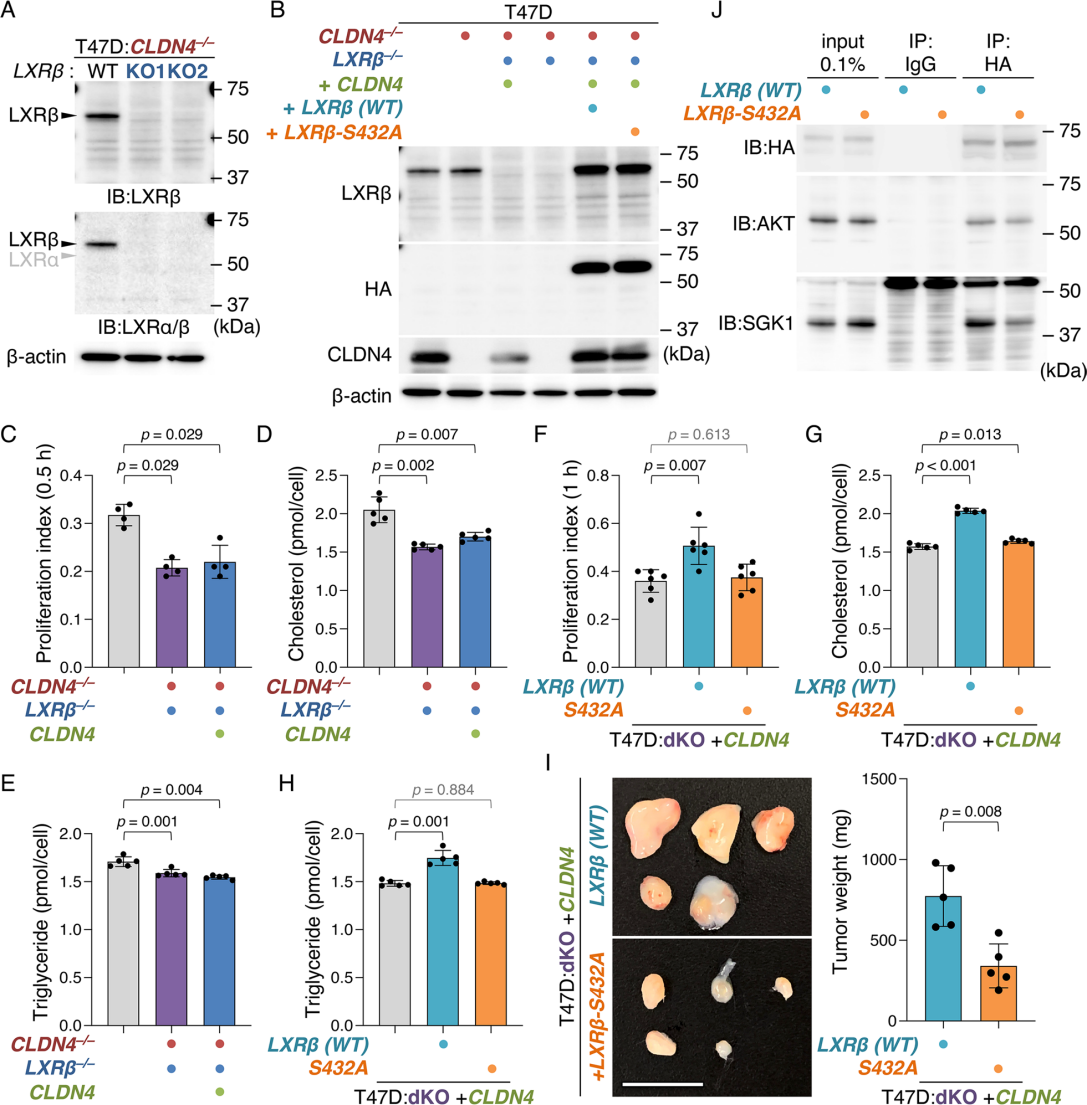
The CLDN4 signaling targets LXRβS432 in breast cancer cells. **A** Western blot analysis showing the absence of LXRβ protein in T47D:*CLDN4*^*–/–*^*:LXRβ*^*–/–*^ cells. **B** Western blot analysis for the indicated proteins in the revealed T47D cells. **C**, **F** Quantitative BrdU assay for the indicated cells. The BrdU/DAPI levels are plotted and shown in the histograms (mean ± SD; n = 4 for C; n = 6 for F). 30 min (**C**) and 60 min (**F**) indicate exposure time for BrdU labeling. dKO, *CLDN4*^*–/–*^*:LXRβ *^*–/–*^. **D**, **E**, **G**, **H** The intracellular cholesterol (**D**, **G**) and triglyceride (**E**, **H**) levels in the indicated T47D cells are plotted and shown in histograms (mean ± SD; n = 5). **I** Gross appearance and weight of the indicated xenografts at 28 days after the inoculation. The tumor weight is plotted and shown in histograms (mean ± SD; n = 5). Similar results were obtained from xenograft experiments using different clones. Scale bar, 1 cm. **J** Association of either AKT or SGK1 and LXRβ in 293T cells transiently transfected with the *HA-LXRβ -WT* or *HA-LXRβ -S432A* expression vector. In the input lanes, 0.1% of the input protein samples were loaded. IP, immunoprecipitation; IB, immunoblot

To evaluate whether the CLDN4 signaling drives breast cancer metabolism and progression via LXRβ, we generated T47D:*CLDN4*^*–/–*^*:LXRβ*^*–/–*^ (hereafter designated as "T47D:dKO"), T47D:dKO:*CLDN4,* and T47D:dKO:*CLDN4:LXRβ* cells (Fig. 5A, B; Additional file 1: Fig. S2B). As mentioned above, the AKT-consensus phosphorylation motifs are conserved in 14 of 48 members of human nuclear receptors, including LXRβ (RXXS, aa 429 to 432) [45]. Therefore, we also established T47D:dKO:*CLDN4:LXRβS432A* cells, in which LXRβS432 was substituted for an alanine residue, and compared their phenotypes with those in T47D:dKO:*CLDN4:LXRβ* cells. The morphological appearance of these cell lines was similar to that in parental T47D cells (Additional file 1: Fig. S3A). As expected, cell growth, migration, and intracellular levels of cholesterol and triglyceride were significantly decreased in T47D:dKO cells compared with those in parental T47D cells (Fig. 5C–E; Additional file 1: Fig. S9A). In addition, the rescue of the CLDN4 expression in T47D:dKO cells failed to increase cell proliferation and migration, as well as intracellular cholesterol and triglyceride concentrations, indicating that LXRβ is absolutely required to stimulate these CLDN4-initiated cellular events. Importantly, CLDN4-enhanced cell proliferation and migration, as well as cholesterol homeostasis and fatty acid metabolism, were significantly diminished in T47D:dKO:*CLDN4:LXRβS432A* cells (two clones) compared with those in T47D:dKO:*CLDN4:LXRβ* cells (Fig. 5F–H; Additional file 1: Fig. S9B, C). Moreover, the tumor growth in T47D:dKO:*CLDN4:LXRβS432A* xenograft was significantly reduced than that in T47D:dKO:*CLDN4:LXRβ* cells (Fig. 5I). Taken collectively with our data showing that the level of LXRβ protein in T47D:dKO:*CLDN4:LXRβS432A* cells was similar to that in T47D:dKO:*CLDN4:LXRβ* cells (Fig. 5B), these results revealed that LXRβS432A is critical for the CLDN4-provoked breast cancer metabolism and advancement. Note also that AKT and SGK1 (serum- and glucocorticoid-regulated kinase), which shares a high degree of homology and the same consensus phosphorylation motif, were associated with transiently introduced LXRβ in 293T cells (Fig. 5J). Both kinases formed a complex not only with WT LXRβ but also with LXRβS432A in 293T cells, suggesting that they also bind to LXRβ at different consensus phosphorylation sites from S432.

We next verified cell viability in T47D and T47D:*CLDN4*^*–/–*^ cells grown in the presence or absence of cholesterol. The viable cell numbers after cholesterol treatment were significantly increased in both T47D and T47D:*CLDN4*^*–/–*^ cells (Additional file 1: Fig. S10A). It is noteworthy that, upon cholesterol addition, the relative cell numbers in T47D:dKO:*CLDN4:LXRβS432A* cells were also significantly elevated, and the increased level was similar to that in T47D:dKO:*CLDN4:LXRβ* cells (Additional file 1: Fig. S10B), suggesting that the CLDN4–LXRβS432 signaling regulates breast cancer cell proliferation by increasing the intracellular cholesterol levels. In addition, a synthetic LXR ligand T0901317 decreased the viable cell numbers of T47D, T47D:dKO:*CLDN4:LXRβ*, T47D:*CLDN4*^*–/–*^, and T47D:dKO:*CLDN4:LXRβS432A* cells, in the latter two of which the effects of T0901317 were weak compared with those in the former two (Additional file 1: Fig. S11).

### The CLDN4 signaling LXRβ-dependently and independently controls gene expression in breast cancer cells

**Fig. 6:**
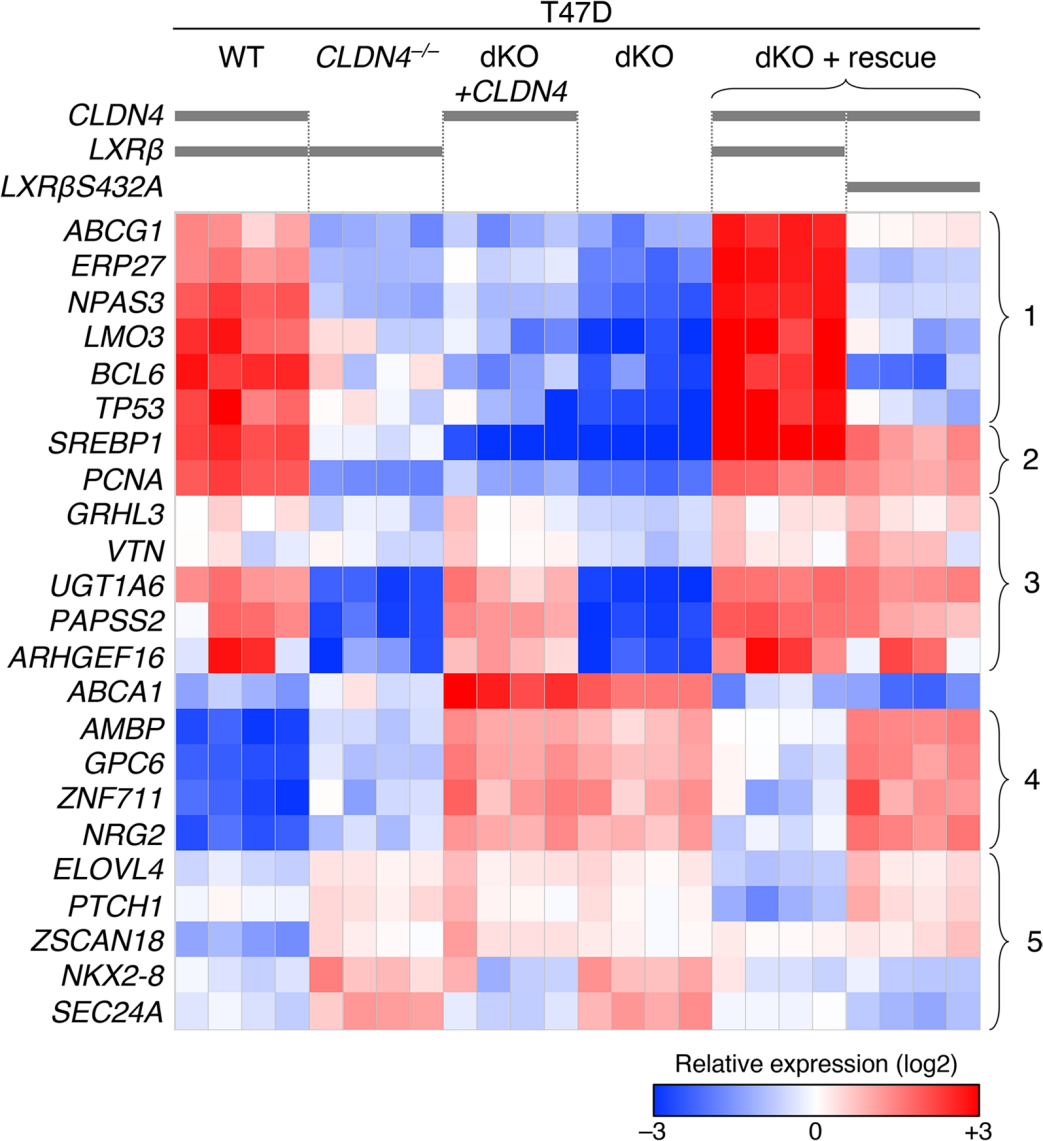
The CLDN4 signaling LXRβ-dependently and independently regulates gene expression in breast cancer cells. RT-qPCR analysis for the indicated genes was performed in the revealed T47D cell lines. The expression levels relative to *GAPDH* are shown in the heatmap. dKO, *CLDN4*^*–/–*^*:LXRβ*^*–/–*^

To categorize downstream genes whose expression levels are altered by the CLDN4 signaling, we next compared, by RT-qPCR, the expression of 23 genes, which gene products are known to be associated with malignant phenotypes, in T47D, T47D:*CLDN4*^*–/–*^, T47D:dKO, T47D:dKO:*CLDN4*, T47D:dKO:*CLDN4:LXRβ*, and T47D:dKO:*CLDN4:LXRβS432A* cells (Fig. 6). Among these CLDN4-regulating genes, the expression of 8 genes (clusters #1 and #2) was induced via LXRβ, and 6 of these eight genes appeared to be upregulated in an LXRβS432-dependent manner (clusters #1). On the other hand, five genes (cluster #3) were activated by the LXRβ-independent CLDN4 signaling. In addition, the expression of 5 genes (cluster #4) was weakly suppressed by the LXRβ-independent CLDN4 signaling and strongly inhibited in an LXRβS432-dependent fashion. Furthermore, the expression of 5 genes (cluster #5) seemed to be repressed by both LXRβS432-dependent and independent CLDN4 signalings. Additionally, the *ABCA1* (ATP-binding cassette subfamily A member 1) expression was suppressed by the LXRβ-dependent and LXRβS432-independent CLDN4 signaling. It should also be noteworthy that the CLDN4–LXRβ signaling appeared to regulate the expression of *ABCA1*, *ABCG1*, and *SREBP1* (Sterol regulatory element-binding transcription factor 1) genes among analyzed genes involved in cholesterol and fatty acid homeostasis.

### TNBC cases highly expressing both CLDN4 and LXRβ reveal a poor outcome

Using The Cancer Genome Atlas (TCGA), we subsequently checked the expression of *LXRα* and *LXRβ* genes in breast cancer cases. As shown in Additional file 1: Fig. S12A, *LXRβ* mRNA was predominantly expressed in 96.4% of these breast cancer tissues. Dominant expression of *LXRβ* transcripts was confirmed by relative *LXRβ/α* amount in individual subjects (Additional file 1: Fig. S12B). We then verified by immunohistochemistry the expression of LXRα and LXRβ protein in 24 cases of breast cancer. LXRα protein was obviously expressed in CD68-positive macrophages of the lymph node as internal controls (Additional file 1: Fig. S13A), whereas it was not detected in any breast cancer tissues (Additional file 1: Fig. S13B). In contrast, LXRβ protein was observed in most cases of breast cancer tissues (Additional file 1: Fig. S13C). Taken together with our findings concerning the LXRα/β expression in T47D, MCF-7, and MDA-MB-231 cells, we concluded that LXRβ is the overwhelmingly dominant subtype of LXRs in breast cancer at the protein levels.

**Fig. 7:**
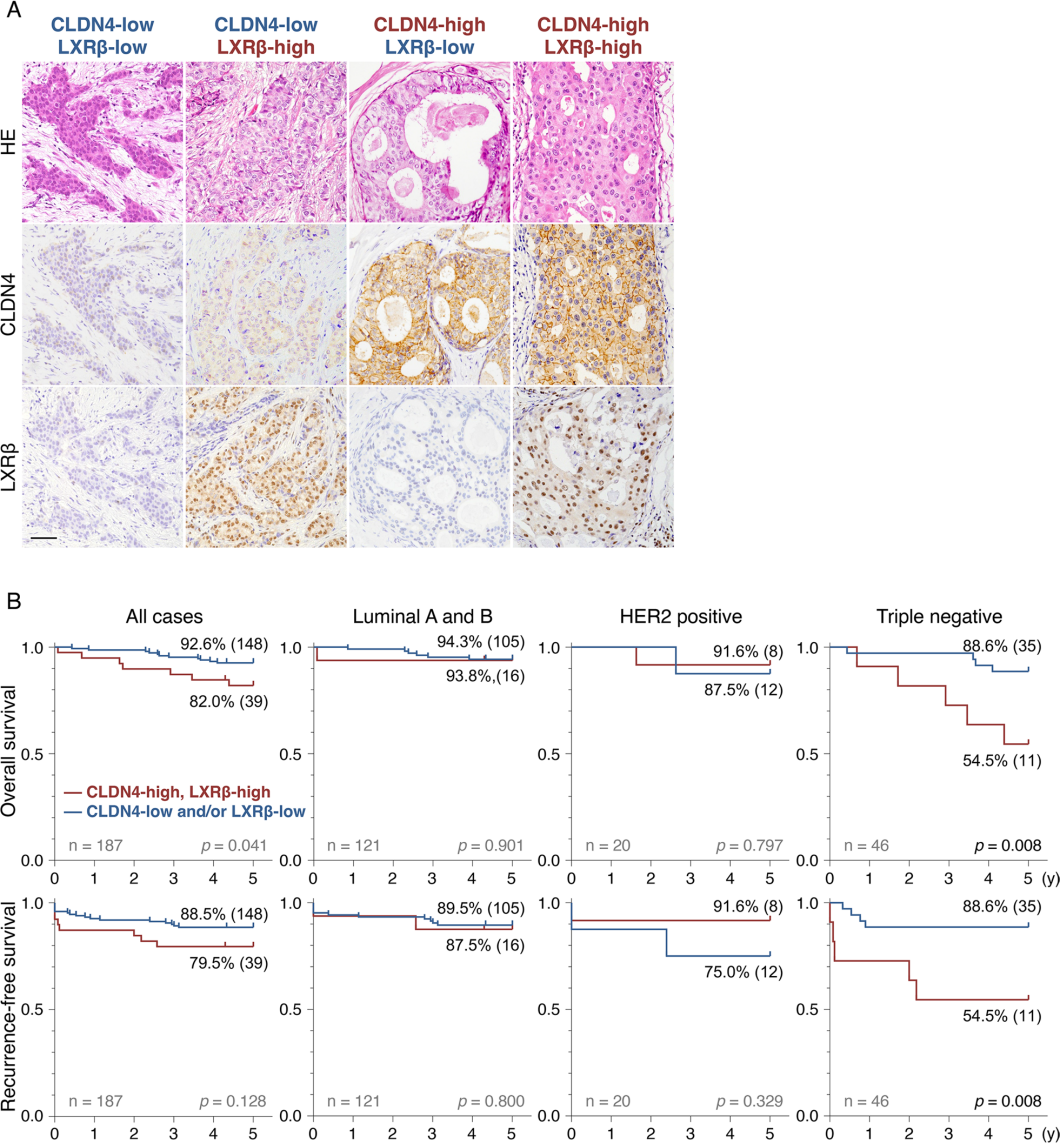
The "CLDN4-high/LXRβ-high" TNBC cases exhibit poor prognosis. **A** Immunohistochemical staining of CLDN4 and LXRβ in breast cancer tissues. Four representative patterns are presented depending on the expression levels of CLDN4 and LXRβ. HE, hematoxylin–eosin. Scale bar, 100 µm. **B** The overall and recurrence-free survival in "CLDN4-high/LXRβ-high" and "CLDN4-low and/or LXRβ-high" groups of the indicated breast cancer subjects

We next determined, by immunohistochemistry, the expression of both CLDN4 and LXRβ in breast cancer tissues resected from the 187 patients (*SI Appendix*, Additional file 1: Table S1). Using the immunoreactive score [45] (for CLDN4) and Allred score [46] (for LXRβ), we semi-quantified the CLDN4 and LXRβ expression (Additional file 1: Fig. S14). Based on the Receiver-Operating Characteristic (ROC) analysis, we divided the subjects into four groups: CLDN4-low/LXRβ-low, CLDN4-high/LXRβ-low, CLDN4-low/LXRβ-high, and CLDN4-high/LXRβ-high (Fig. 7A; Additional file 1: Fig. S15A, B).

On Kaplan–Meier plots for 187 breast cancer cases, the overall survival rate in the "CLDN4-high/LXRβ-high" group was slightly lower than that in the "CLDN4-low and/or LXRβ-low" group, whereas these two groups did not possess significant differences in recurrence-free survival (Fig. 7B). Importantly, however, the "CLDN4-high/LXRβ-high" group in TNBC cases revealed significantly shorter overall and recurrence-free survival than the "CLDN4-low and/or LXRβ-low" group. The 5-year recurrence-free survival in the "CLDN4-high/LXRβ-high" and the "CLDN4-low and/or LXRβ-low" groups were 54.5% and 88.6%, respectively. By contrast, in luminal A/B and HER2-positive breast cancer cases, there were no significant differences in overall and recurrence-free survival between "CLDN4-high/LXRβ-high" and "CLDN4-low and/or LXRβ-low" groups, possibly due to the influence of valid endocrine therapy and anti-HER2 drug, both of which could mask effects of the CLDN4–LXRβ signaling in breast cancer.

## Discussion

In the present study, we demonstrated that CLDN4 accelerates breast cancer progression in vitro and in vivo. This was apparent because KO of the human *CLDN4* gene led to a reduction in cell proliferation, migration, and invasion in two distinct breast cancer cell lines T47D and/or MCF-7. Conversely, these malignant phenotypes were stimulated by the re-expression of CLDN4 in T47D:*CLDN4*^*–/–*^ and MCF-7:*CLDN4*^*–/–*^ cells, as well as by the introduction of the *CLDN4* gene in MDA-MB-231 cells. In addition, using T47D:*CLDN4*^*–/–*^ and MDA-MB-231:*CLDN4* xenografts, it was shown that CLDN4 promotes the tumor growth and cell proliferation of breast cancer cells in vivo. Tumor budding, small clusters of cancer cells, was hindered in T47D:*CLDN4*^*–/–*^ xenografts compared with T47D xenografts, further indicating that CLDN4 functions as a tumor promoter in breast cancer cells. These results are consistent with those of a previous report, which showed that CLDN4 stimulates malignant phenotypes in MCF-7 cells [47].

We also showed that CLDN4 activates SFK and the downstream AKT in breast cancer cells in the EC2- and Y197-dependent manners, leading to promote their cell proliferation. This conclusion was drawn from the following results: 1) colocalization of CLDN4 and pSFK along cell boundaries was apparently observed in both T47D and MCF-7 cells, whereas it was diminished in T47D:*CLDN4*^*–/–*^ and MCF-7:*CLDN4*^*–/–*^ cells, as well as in C-CPE-treated T47D and MCF-7 cells; 2) the pSFK levels were decreased and increased in T47D:*CLDN4*^*–/–*^ and MDA-MB-231:*CLDN4* cells compared with their parental cells, respectively; 3) the pSFK intensities in T47D:*CLDN4*^*–/–*^*:CLDN4ΔEC2* and T47D:*CLDN4*^*–/–*^*:CLDN4ΔC* cells were lower than those in T47D:*CLDN4*^*–/–*^*:WT-CLDN4* cells; 4) the pSFK levels in T47D:*CLDN4*^*–/–*^*:CLDN4Y197A* cells were reduced compared to those in T47D:*CLDN4*^*–/–*^*:WT-CLDN4* and T47D:*CLDN4*^*–/–*^*:CLDN4Y193A* cells; 5) the CLDN4-triggered cell proliferation was reversed in T47D:*CLDN4*^*–/–*^*:CLDN4ΔEC2*, T47D:*CLDN4*^*–/–*^*:CLDN4ΔC,* and T47D:*CLDN4*^*–/–*^:*CLDN4Y197A* cells compared with that in T47D:*CLDN4*^*–/–*^*:WT-CLDN4* cells; 6) The CLDN4-provoked cell proliferation was prevented in MDA-MB-231:*CLDN4ΔC* cells compared with MDA-MB-231:*CLDN4* cells; 7) the CLDN4-initiated cell proliferation abrogated upon C-CPE, PP2 and AKT inhibitor VIII treatment in T47D cells but not in T47D:*CLDN4*^*–/–*^ cells. We have recently reported that the EC2 domain and Y196/200 of CLDN6 are required to recruit and activate SFKs and to stimulate malignant phenotypes of endometrial cancer cells [44, 47]. Thus, at least two CLDN subtypes propagate SFKs by similar mechanisms, namely in the EC2- and the C-terminal tyrosine residue-dependent manners. Since CLDN4Y197 and CLDN6Y200 are conserved in human and mouse CLDN1/2/5/9/17/18 [19], it would be interesting to determine the biological significance of the corresponding tyrosine residues in various types of cancer.

Our RNA sequencing analysis, using T47D and T47D:*CLDN4*^*–/–*^ cells as well as MCF-7 cells and MCF-7:*CLDN4*^*–/–*^ cells, first suggested a link between CLDN4 and LXRs signalings in breast cancer cells. We also showed that LXRβ but not LXRα protein is expressed in T47D, MCF-7, and MDA-MB-231 cells, as well as in breast cancer analyzed. Therefore, we subsequently generated T47D:*CLDN4*^*–/–*^*:LXRβ*^*–/–*^ (T47D:dKO) and a series of rescue cell lines. Consequently, by comparing phenotypes in T47D:dKO:*CLDN4* cells with those in T47D:dKO cells, we demonstrated that the CLDN4 signaling enhances cell growth, migration, and intracellular cholesterol and triglyceride levels via LXRβ. LXRs play a key role not only in maintaining cholesterol homeostasis and fatty acid metabolism but also in cellular proliferation [48]. Taken together with the findings showing that LXRα controls the growth of skin and oral squamous cell carcinoma by altering the cholesterol homeostasis [49, 50], our results suggest that the CLDN4 signaling promotes cell proliferation in breast cancer cells, possibly by LXRβ-mediated control of genes involved in cancer metabolism. More importantly, CLDN4-enhanced cell proliferation, migration, and tumor growth, as well as intracellular levels of cholesterol and triglyceride, were prevented in T47D:dKO:*CLDN4:LXRβS432A* cells compared with that in T47D:dKO:*CLDN4:LXRβ* cells, indicating that LXRβS432 is responsible for the CLDN4/SFK/AKT-accelerated breast cancer metabolism and progression. Furthermore, AKT and SGK1 formed a complex with LXRβ in 293T cells, reinforcing the conclusion. We previously demonstrated that the CLDN6/SFK/AKT signaling directs S379 and S518 in mouse RARγ and human ERα, respectively [18, 19, 39]. Hence, these results revealed that the cell-adhesion signaling targets the AKT-consensus phosphorylation sites in at least three nuclear receptors.

Another conclusion of our study is that the CLDN4 signaling LXRβ-dependently and independently regulates a range of gene expressions in breast cancer cells. RT-qPCR analysis, using T47D, T47D:*CLDN4*^*–/–*^, T47D:dKO, T47D:dKO:*CLDN4*, T47D:dKO:*CLDN4:LXRβ*, and T47D:dKO:*CLDN4:LXRβS432A* cells, uncovered that the CLDN4-controlled genes are categorized into at least six groups in terms of distinct requirement of LXRβ and LXRβS432. Among 23 CLDN4-regulating genes whose products are associated with tumor progression in various cancers, eight genes were upregulated via LXRβ, six of which were activated in an LXRβS432-dependent manner. Because both LXRβ and LXRβS432 were essential for the CLDN4-accelerated cell proliferation and tumor growth in breast cancer cells, the LXRβ- and LXRβS432-dependent CLDN4-controlling gene products would be critical to promote breast cancer progression.

Clinicopathologically, we found that the "CLDN4-high/LXRβ-high" and "CLDN4-low and/or LXRβ-low" TNBC subjects possess poor and relatively favorable outcomes, respectively. This is reasonable because a series of our analyses disclosed that the CLDN4 signaling stimulates breast cancer progression through LXRβ. Thus, evaluating expression levels of both CLDN4 (input signal) and LXRβ (output signal) is required to predict distinct prognoses in TNBC cases. Along this line, clinicopathological analysis using expression levels of either CLDN4 [35–37] or LXRβ [51] does not seem to be enough to predict a prognosis in breast cancer, especially in TNBC. Since there is no valid medication for TNBC, it should also be worth noting that the CLDN4/LXRβ axis may be a promising therapeutic target for TNBC. For instance, an LXR inverse agonist is effective against various types of cancer without obvious side effects [52]; therefore, it should be determined whether the LXRβ-targeting treatment could be a therapeutic option in the "CLDN4-high/LXRβ-high" TNBC cases.

## Conclusions

In summary, the present study highlighted that the CLDN4-based cell adhesion signaling accelerates breast cancer metabolism and advancement via LXRβ, especially through LXRβS432. We also demonstrated that high expression of both CLDN4 and LXRβ predicts poor prognosis in TNBC patients. Further study is required to verify whether a similar link between cell adhesion and transcription factor signalings coordinates diverse physiological and pathological events, including tumor metabolism and advancement in various types of cancers.

## Methods

### Antibodies

The antibodies used in this study are listed in Additional file 1: Table S2.

### Cell lines and cell culture

Breast cancer cell lines T47D (HTB-133) and MDA-MB-231 (HTB-26TM) were purchased from the American Type Culture Collection (ATCC). MCF-7 (RCB1904) and SKBR-3 (RCB2132) were obtained from RIKEN Bioresource Center. These cell lines were maintained in Dulbecco's Modified Eagle Medium (DMEM) with 10% fetal bovine serum (FBS; Sigma-Aldrich) and 1% penicillin–streptomycin-amphotericin B suspension (161-23181, FUJIFILM). For assays, the cells were grown in a phenol red-free medium with charcoal-treated FBS to exclude fat-soluble ligands. For the preparation of charcoal-treated FBS, 500 ml of FBS was treated with 0.5 g of charcoal dextran-coated (Sigma) overnight at 4ºC, followed by filtration using 0.22 µm cellulose acetate filter membranes. The cells were treated for 24 h with 1 µg/ml of C-CPE, 10 µM of PP2 (529573, Sigma-Aldrich), 0.1 µM of AKT inhibitor VIII (CS-0001, Funakoshi), 1 mg/ml of Cholesterol-Water Soluble (C4951-30MG; Sigma-Aldrich), and 1–25 µM of T0901317 (71810, Cayman Chemical) 24 h after plating. C-CPE production and purification were performed as described previously by using *E.coli* BL21 and the expression vector pET16b coding C-CPE194-319 [42].

### Genome editing

We used the CRISPR technique to establish the *CLDN4* and *LXRβ (NR1H2)* knockout cell lines. Annealed oligos, including targets described in Additional file 1: Fig. S2, were cloned into the *Esp3*I site of lentiCRISPR v2 plasmid (#52961 Addgene). Although lentiCRISPR v2 was originally designed to be packaged into lentivirus, the plasmids were directly and transiently transfected into the parental cells by Lipofectamine 3000 (15292465, Thermo Fisher Scientific) in the present study. Twelve h after transfection, the cells were exposed to 10 µg/ml of puromycin for 24 h, followed by limiting dilution and genotyping by genomic PCR. Knockout of *CLDN4* and *NR1H2* genes was verified by DNA sequencing after TA-cloning of genomic PCR products.

### Expression vectors, transfection, and establishment of stable cell lines

The protein-coding regions of human *CLDN4* or *NR1H2* were cloned into the *Not*I/ *BamH*I site of the CSII-EF-MCS-IRES2-Venus (RDB04384, RIKEN) plasmid. Expression vectors of mutant genes, including CLDN4ΔEC2, CLDN4ΔC, CLDN4Y193A, CLDN4Y197A, and LXRβS432A, were established using a standard PCR-based site-directed mutagenesis protocol. The overexpression or rescued cell lines were established by lentiviral transfection. First, lentiviral vectors were generated by transfecting 1.0 × 10^7^ cells of 293 T with 10 µg of the CSII plasmids containing the target genes, 5 µg of packaging plasmids psPAX2 (#12260, Addgene), and pCMV-VSV-G (#8454, Addgene) using Polyethylenimine Max (PEI Max; 24765-1, Cosmo Bio). Culture media containing recombinant lentiviruses were collected 72 h after transfection and directly added to the cell culture medium of T47D:*CLDN4*^*–/–*^, T47D:*CLDN4*^*–/–*^*:LXRβ*^*–/–*^ (T47D:dKO), MCF-7:*CLDN4*^*–/–*^, and MDA-MB-231 cells. After more than 7 days and three times passages, the cells were used for further analysis. T47D:dKO*:CLDN4*, T47D:dKO*:CLDN4:LXRβ*, T47D:dKO*:CLDN4:LXRβS432A* cells were single-cell cloned by limiting dilution.

### Cell proliferation, migration, invasion, and apoptosis assays

Cell proliferation index was evaluated by incorporation of bromodeoxyuridine (5-Bromo-2-DeoxyUridine, BrdU; 19–960, Sigma-Aldrich). 24–48 h after passage, cells were exposed to BrdU for 30 or 60 min. The specimens were fixed with 4% paraformaldehyde and 0.1% Triton-X, followed by immunostaining with the anti-BrdU antibody and its standard protocol.

Total viable cell counts were quantified by CellTiter 96 AQueous One Solution Cell Proliferation Assay (MTS) Kit (G3582, Promega). One thousand cells were seeded on 96-well plates, and the reagent was added to each well after 48 h, followed by measurement of absorbance at 490 nm.

To evaluate cell migration, wound areas were generated by scratching with disposable 200 µl pipette tips 48 h after passage. Photographs of the wound areas were taken at the same locations after scratching by the indicated intervals, using a phase-contrast microscope. Wound healing was calculated as the percentage of the remaining cell-free area compared with the initial wound area using ImageJ software (Wayne Rasband National Institutes of Health).

BioCoat Matrigel Invasion Chamber (#354480, Corning) was used for assessing cell invasion by following the provider's protocol. Briefly, 2.0 × 10^4^ cells were transferred to each well. Twenty-four h after passage, the samples were fixed with 100% methanol and stained with crystal violet. The invasion index was calculated by dividing by cell numbers in negative control membranes, which consist of empty mesh but do not contain matrigel.in situ Cell Death Detection Kit (11684795910, Sigma-Aldrich) was used for the evaluation of cell apoptosis.

### Measurement of cholesterol and triglyceride content

Cholesterol and triglyceride in cell lysates were measured by Cholesterol/Cholesterol Ester-Glo Assay (J3190, Promega) and Triglyceride-Glo Assay (J3160, Promega), respectively. One thousand cells were plated in 96-well plates, and the cholesterol and triglyceride contents were quantified by the kits following the manufacturer’s protocols after 24 h.

### Xenograft model

Xenograft studies were performed in 8-week-old CB17/IcrJcl-*Prkdc*^*scid*^ female mice (CLEA Japan). 5.0 × 10^6^ cells were subcutaneously injected into the back of anesthetized mice. Twenty-eight days after injection, the mice were ethically sacrificed, and tumor tissues were collected. The samples were immediately fixed with 10% neutral buffered formalin solution for 24–36 h. Hematoxylin–eosin staining and Ki-67 staining were performed following standard protocols optimized for human tissues. Ki-67 index was assessed in the hot spot of each tumor. Tumour budding was analyzed by counting tumor clusters consisting of two to six cells at the five high-power fields of invasion fronts of each specimen.

### Immunoprecipitation and immunoblot

Total cell extracts were collected by using CellLytic MT Cell Lysis Reagent (C3228, Sigma-Aldrich) and were subsequently sonicated with three or four bursts of 5–10 s. Immunoprecipitation was performed using Immunoprecipitation Kit Protein G (11719386001, Sigma-Aldrich), following the manufacturer's protocol. 1 µg of ChromPure Rat IgG (012-000-003, Jackson Immunoresearch Laboratories) was used as a negative control. Whole-cell lysates or immunoprecipitated samples were mixed with sample loading buffer containing 2-mercaptoethanol and incubated for 10 min at 95 °C. They were resolved by one-dimensional SDS-PAGE and electrophoretically transferred onto a polyvinylidene difluoride membrane. The membranes were saturated with PBS containing 4% skimmed milk or PVDF Blocking Reagent for Can Get Signal (NYPBR01, TOYOBO) for 30 min. After rinsing in TBS containing 0.1% Tween 20, the membranes were incubated with a primary antibody solution diluted in PBS or Can Get Signal Solution 1 (NKB-101, TOYOBO) for 1 h at room temperature or overnight at 4 °C, followed by 1-h incubation with horseradish peroxidase (HRP)-conjugated secondary antibodies diluted in PBS or Can Get Signal Solution 2 (NKB-101, TOYOBO). An anti-GFP antibody was used for detecting Venus, of which amino-acid alignment completely matches the antigen region of GFP. They were rinsed again and exposed to EzWestLumi One (ATTO). After rinsing with 10% H_2_O_2_ to inactivate HRP, each membrane was hybridized with HRP-conjugated anti-beta actin antibody as loading controls. Total cell extract of F9 cells [53] was used as the positive control shown in Additional file 1: Fig. S1A. Each signal was quantified by ImageJ software (Wayne Rasband National Institutes of Health) and divided by the corresponding actin levels.

### Immunofluorescence and imaging

Cells were grown on coverslips coated with Cellmatrix Type I-A (Nitta gelatin). The samples were fixed in 4% paraformaldehyde and 0.2% Triton-X for ten min at room temperature. After washing with PBS, they were preincubated in PBS containing 5% skimmed milk. They were subsequently incubated overnight at 4 °C with primary antibodies diluted in Signal Booster Immunostain F (BCL-ISF, Beacle), then rinsed again with PBS, followed by a reaction for 1 h at room temperature with appropriate secondary antibodies. All samples were examined using a laser-scanning confocal microscope (FV1000, Olympus). Photographs were processed with Photoshop CC (Adobe) and ImageJ software (Wayne Rasband National Institutes of Health).

### RNA extraction, RT-PCR, and RNA sequencing

Total RNA was isolated from cells using TRIzol RNA Isolation Reagents (15596018, Thermo Fisher Scientific). For RT-qPCR, reverse transcription was performed using SensiFAST cDNA Synthesis Kit (BIO-65054, meridian BIOSCIENCE), and target genes were quantified by THUNDERBIRD SYBR qPCR Mix (QPS-201, TOYOBO) and Step One Real-Time PCR System (Applied Biosystems) using the primers listed in Additional file 1: Table S3. The expression levels of the target genes were normalized to the corresponding *GAPDH* expression.

RNA sequencing and mapping were performed by BINDS, a platform project for supporting drug discovery and life science research in Japan. To generate mapped bam files, the index-trimmed single-end 100 bp reads were aligned to the human reference genome (GRCh38 v90). The mapped bam files were imported to SeqMonk software (Babraham Bioinformatics) as single-ended RNA-Seq data. Then they were quantitated by using a standard RNA-Seq quantitation pipeline consisting of TopHat2, CuffLinks2, and CummeRbund. Raw data was uploaded to Gene Expression Omnibus (https://www.ncbi.nlm.nih.gov/geo/) as GSE207704.

### TCGA expression analysis

Gene expression and clinical data of 1100 breast cancer cases in TCGA cohorts were downloaded from cBioPortal (www.cbioportal.org/). mRNA expression levels of *LXRα (NR1H3)* and *LXRβ (NR1H2)* were imported as RSEM values [53] and visualized as a scatter plot by Prism 9 (GraphPad).

### Tissue collection and immunohistochemistry

Formalin-fixed paraffin-embedded (FFPE) tissue sections were obtained from 187 patients with breast cancer (age, 27–85 years; average ± SD = 55.7 ± 11.7) who underwent a total or partial mastectomy and sentinel lymph node biopsy or axillary lymph node dissection between 2008 and 2013 at Fukushima Medical University Hospital (Additional file 1: Table S1). The subjects were limited to patients who were confirmed to have at least 5-year outcomes. Detailed information, including postoperative pathology diagnosis reports, age, stage (The UICC TNM classification), histological type, ER, PgR, HER2, recurrence status, recurrence-free survival, and overall survival, was obtained. Patients' backgrounds were anonymized. CLDN4 and LXRβ expression were independently and blindly evaluated by two pathologists and one breast specialist. The signal intensity of CLDN4 was semi-quantified using the Immunoreactive Score (IRS; Additional file 1: Table S4) [45], and the lowest scores were adopted. Briefly, staining intensity (SI) was classified into four levels (0, negative; 1, weak; 2, moderate; 3, strong) and staining range (percentage of positive cells; PP) into five levels (0, < 1%; 1, 1–10%; 2, 11–30%; 3, 31–50%; 4, > 50%). IRS was calculated by multiplying SI and PP. Scores 0–4 were defined as CLDN4-low, and scores 6–12 as CLDN4-high. On the other hand, LXRβ expression was evaluated by the Allred Score, which is used for assessing the ER and PgR in the breast cancer [46], and the highest scores were adopted. Scores 0–6 were defined as LXRβ-low and scores 7–8 as LXRβ-high.

### Statistical analysis

Statistical significance for cell proliferation was analyzed by the Mann–Whitney test, while those for cell migration, invasion, and growth of xenograft were analyzed by Welch's t-test. GSEA was performed using GSEA v4.2.3 software and hallmark gene sets which are publically available from the Broad Institute [54]. Kaplan–Meier method was used for survival analyses, and differences between groups were analyzed using the log-rank test. Two-tailed *p* values < 0.05 were considered to indicate a statistically significant result when comparing two groups. Benjamini and Hochberg's correction method was used to counteract the multiple comparisons problem when comparing more than three groups. All statistical analyses were performed using GraphPad Prism v9.4.0 software.

## Supplementary Information

**Additional file 1: Fig. S1**. Expression of CLDN4 in human breast cancer cell lines. (A and B) Western blot (A) and confocal images (B) for the indicated proteins in MCF-7, T47D, SKBR-3, and MDA-MB-231 cells. Mouse F9 embryonal carcinoma cells are used as a positive control. Scale bars, 20 μm. **Fig. S2**. Knockout (KO) of the *CLDN4* and *LXRβ* genes in human breast cancer cell lines using the CRISPR/Cas9 method. (A) KO of the *CLDN4* gene in T47D:*CLDN4*^*–/–*^ and MCF-7:*CLDN4*^*–/–*^ cells is confirmed by DNA sequencing. (B) KO of the *LXRβ* gene in T47D:*CLDN4*^*–/–*^*:LXRβ *^*–/–*^ cells are verified by DNA sequencing. **Fig. S3. Phase-contrast images in the indicated cell lines. (**A and B) Representative images of each wild-type (WT) or transgenic T47D (A) and MCF-7 (B) cell line are shown. Scale bars, 100 μm. **Fig. S4**. CLDN4 enhances cell invasion in the breast cancer cell line T47D. (A and B) Representative and quantitative invasion assay for the indicated cells. The invasion index is plotted and shown in the histograms (mean ± SD; *n* = 5). (C) The absence of CLDN4 does not affect apoptosis in T47D cells. Cells are subjected to TUNEL assay together with DAPI staining. Scale bars, 100 μm. **Fig. S5**. CLDN4 accelerates malignant activities of the breast cancer cell line MDA-MB-231. (A) Western blot analysis indicating overexpression of CLDN4 protein in MDA-MB-231:*CLDN4* cells. (B) BrdU assay for the indicated cells. The BrdU/DAPI levels are plotted and shown in the histograms (mean ± SD; *n* = 6). (C) Wound healing assay of the indicated cells. The wound closure rates are plotted and shown in the histograms (mean ± SD; *n* = 20). (D) Invasion assay for the indicated cells. The invasion index is plotted and shown in the histograms (mean ± SD; *n* = 5). **Fig. S6**. The C-terminal cytoplasmic domain of CLDN4, SFK, and AKT are involved in the CLDN4-accelerated breast cancer proliferation. T47D and T47D:*CLDN4*^*–/–*^ cells were grown for 24 h in the presence of vehicle, C-CPE (C-terminal half of *Clostridium Perfringens* enterotoxin; 1 μg/ml), the SFK inhibitor PP2 (10 μM), or the AKT inhibitor AKT inhibitor VIII (0.1 μM). The BrdU/DAPI levels are shown in histograms (mean ± SD; *n* = 6). **Fig. S7**. RNA sequence analysis showing that the CLDN4 signaling controls the expression of various genes in breast cancer cells. Genes whose expression was significantly down- or up-regulated in both T47D:*CLDN4*^*–/–*^ and MCF-7:*CLDN4*^*–/–*^ cells compared with their parental cells (*p* < 0.05) are shown in the heatmap. Two batches of each cell line were subjected to RNA sequence analysis, and the average of fold changes of the revealed genes is indicated. **Fig. S8**. LXRβ is predominantly expressed in MCF-7, T47D, and MDA-MB-231 cells. (A) RT-qPCR analysis for expression of *LXRα* and *LXRβ* mRNA. *LXRα*- or *LXRβ*-expressing 293 T cells are used for positive controls, and the expression levels relative to *GAPDH* are shown as 1. The values are plotted, and the average is indicated as bars. (B) Western blot analysis for the indicated proteins in the revealed cells. A mixture of whole-cell extracts from 293 T cells overexpressing LXRα and LXRβ is used as a positive control. (C) Confocal images of the indicated proteins in the revealed cell lines. Scale bar, 50 μm. **Fig. S9**. LXRβS432 is essential for the CLDN4-triggered breast cancer progression. (A and C) Wound healing assay of the revealed T47D cells. The wound closure rates are plotted and shown in the histograms (mean ± SD; *n* = 8). (B) BrdU assay for the indicated T47D cells. The BrdU/DAPI levels are plotted and shown in the histograms (mean ± SD; *n* = 4). dKO, *CLDN4*^*–/–*^*:LXRβ *^*–/–*^. **Fig. S10**. Treatment of T47D:*CLDN4*^*–/–*^ (A) and T47D:dKO:*CLDN4:LXRβS432A* (B) cells with cholesterol recovers cell viability. The indicated T47D cells were grown for 24 h in the presence or absence of 1 mg/ml cholesterol. The relative cell numbers are plotted and shown in histograms (mean ± SD; *n* = 5). dKO, *CLDN4*^*–/–*^*:LXRβ *^*–/–*^. **Fig. S11**. Effect of a synthetic LXR ligand T0901317 on cell viability of T47D, T47D:*CLDN4*^*–/–*^, T47D:dKO:*CLDN4:LXRβ*, and T47D:dKO:*CLDN4:LXRβS432A* cells. The indicated T47D cells were grown for 24 h in the presence or absence of 1, 5, and 25 μM T0901317. The relative cell numbers are plotted and shown in histograms (mean ± SD; *n* = 5). dKO, *CLDN4*^*–/–*^*:LXRβ *^*–/–*^. **Fig. S12**. *LXRβ* mRNA is mainly expressed in breast cancer subjects. (A and B) Expression of *LXRα* and *LXRβ* transcripts in 1100 breast cancer tissues using The Cancer Genome Atlas (TCGA) database. RSEM values of *LXRα* and *LXRβ* (A) and mRNA expression of *LXRβ* relative to *LXRα* (B) are shown as median and interquartile range. **Fig. S13**. LXRβ but not LXRα is observed in breast cancer tissues. (A) Representative immunohistochemical images for CD68 and LXRα in a lymph node of breast cancer patients. HE, hematoxylin–eosin. Scale bar, 50 µm. (B and C) Representative immunohistochemical images for LXRα (B) and LXRβ (C) in breast cancer tissues. Scale bars, 100 µm. **Fig. S14**. Semi-quantification of the CLDN4 and LXRβ expression in the indicated breast cancer subjects. IRS, immunoreactive score. **Fig. S15**. Classification of breast cancer subjects by expression levels of CLDN4 and LXRβ. (A) The CLDN4 and LXRβ scores are shown as a bubble dot. IRS, immunoreactive score. (B) The number of "CLDN4-high/LXRβ-high" and "CLDN4-low and/or LXRβ-low" groups in the revealed breast cancer cases are indicated. **Fig. S16**. Uncropped images for the indicated Western blot. **Table S1**. Clinicopathological characteristics of the patients with breast cancer. **Table S2**. Antibodies. **Table S3**. PCR primers. **Table S4**. Immunoreactive score (IRS).

## Data Availability

Raw RNA sequencing data were uploaded to Gene Expression Omnibus (https://www.ncbi.nlm.nih.gov/geo/) as GSE207704. The datasets used and/or analyzed during the current study are available from the corresponding author upon reasonable request.

## Abbreviations

ABCA1: ATP-binding cassette subfamily A member 1
BrdU: 5-Bromo-2-deoxyuridine
C-CPE: C-terminal half of *Clostridium perfringens* enterotoxin
CLDN: Claudin
CRISPR: Clustered regularly interspaced short palindromic repeats
DKK1: Dickkopf WNT signaling pathway inhibitor 1
DMEM: Dulbecco's modified Eagle medium
EC1: Extracellular loop-1
EC2: Extracellular loop-2
ER: Estrogen receptor
FBP1: Fructose-bisphosphatase 1
FFPE: Formalin-fixed paraffin-embedded
GSEA: Gene set enrichment analysis
HER2: Human epidermal growth factor receptor 2
IRS: Immunoreactive score
KRT80: Keratin 80
LDLRAD4: Low-density lipoprotein receptor class A domain-containing protein 4
LXR: Liver X receptor
PBS: Phosphate-buffered saline
PCR: Polymerase chain reaction
PgR: Progesterone receptor
PVDF: Polyvinylidene fluoride
RARγ: Retinoic acid receptor-γ
SFK: Src-family kinases
TBS: Tris-buffered saline
TNBC: Triple-negative breast cancer
UICC: Union for International Cancer Control
