# Supplementary material for: Claudin-4-adhesion signaling drives breast cancer metabolism and progression via liver X receptor β
Source: Breast Cancer Res. 2023 Apr 14;25:41. doi: 10.1186/s13058-023-01646-z (PMC10105442; doi:10.1186/s13058-023-01646-z)
Supplement: Supplementary file 1 — Additional file 1: Fig. S1. Expression of CLDN4 in human breast cancer cell lines. (A and B) Western blot (A) and confocal images (B) for the indicated proteins in MCF-7, T47D, SKBR-3, and MDA-MB-231 cells. Mouse F9 embryonal carcinoma cells are used as a positive control. Scale bars, 20 μm. Fig. S2. Knockout (KO) of the CLDN4 and LXRβ genes in human breast cancer cell lines using the CRISPR/Cas9 method. (A) KO of the CLDN4 gene in T47D:CLDN4–/– and MCF-7:CLDN4–/– cells is confirmed by DNA sequencing. (B) KO of the LXRβ gene in T47D:CLDN4–/–:LXRβ –/– cells are verified by DNA sequencing. Fig. S3. Phase-contrast images in the indicated cell lines. (A and B) Representative images of each wild-type (WT) or transgenic T47D (A) and MCF-7 (B) cell line are shown. Scale bars, 100 μm. Fig. S4. CLDN4 enhances cell invasion in the breast cancer cell line T47D. (A and B) Representative and quantitative invasion assay for the indicated cells. The invasion index is plotted and shown in the histograms (mean ± SD; n = 5). (C) The absence of CLDN4 does not affect apoptosis in T47D cells. Cells are subjected to TUNEL assay together with DAPI staining. Scale bars, 100 μm. Fig. S5. CLDN4 accelerates malignant activities of the breast cancer cell line MDA-MB-231. (A) Western blot analysis indicating overexpression of CLDN4 protein in MDA-MB-231:CLDN4 cells. (B) BrdU assay for the indicated cells. The BrdU/DAPI levels are plotted and shown in the histograms (mean ± SD; n = 6). (C) Wound healing assay of the indicated cells. The wound closure rates are plotted and shown in the histograms (mean ± SD; n = 20). (D) Invasion assay for the indicated cells. The invasion index is plotted and shown in the histograms (mean ± SD; n = 5). Fig. S6. The C-terminal cytoplasmic domain of CLDN4, SFK, and AKT are involved in the CLDN4-accelerated breast cancer proliferation. T47D and T47D:CLDN4–/– cells were grown for 24 h in the presence of vehicle, C-CPE (C-terminal half of Clostridium Perfringens ente [file 13058_2023_1646_MOESM1_ESM.docx]

Supplementary Information for

**Claudin-4–adhesion signaling drives breast cancer metabolism and progression via liver X receptor β**

Yuko Murakami-Nishimagi, Kotaro Sugimoto, Makoto Kobayashi, Kazunoshin Tachibana, Manabu Kojima, Maiko Okano, Yuko Hashimoto, Shigehira Saji, Tohru Ohtake, Hideki Chiba

^*^Correspondence: sugikota@fmu.ac.jp and hidchiba@fmu.ac.jp

This file includes:

Figures S1 to S16

Tables S1 to S4

**
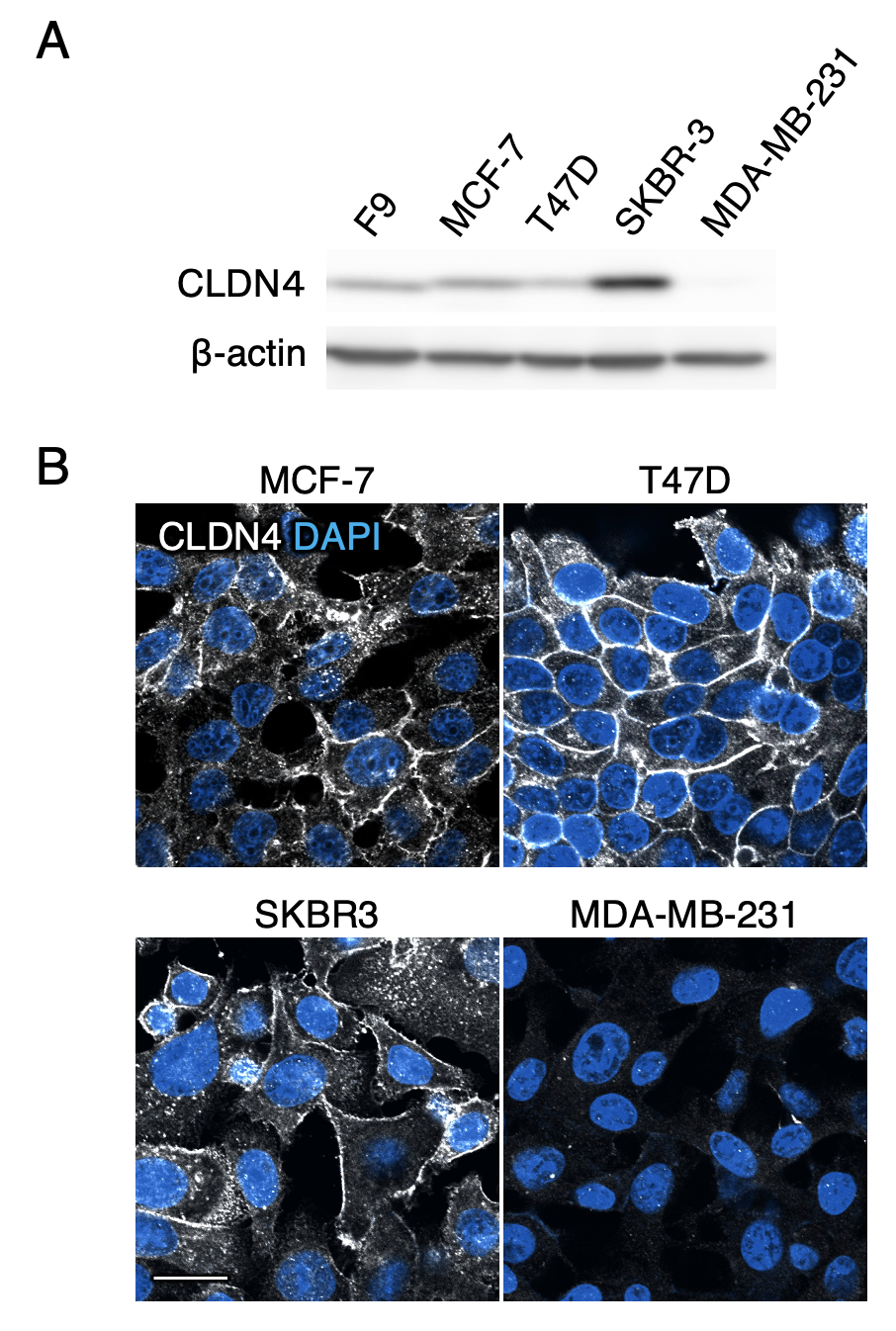
**

Fig. S1. **Expression of CLDN4 in human breast cancer cell lines.** (A and B) Western blot (A) and confocal images (B) for the indicated proteins in MCF-7, T47D, SKBR-3, and MDA-MB-231 cells. Mouse F9 embryonal carcinoma cells are used as a positive control. Scale bars, 20 μm.

**
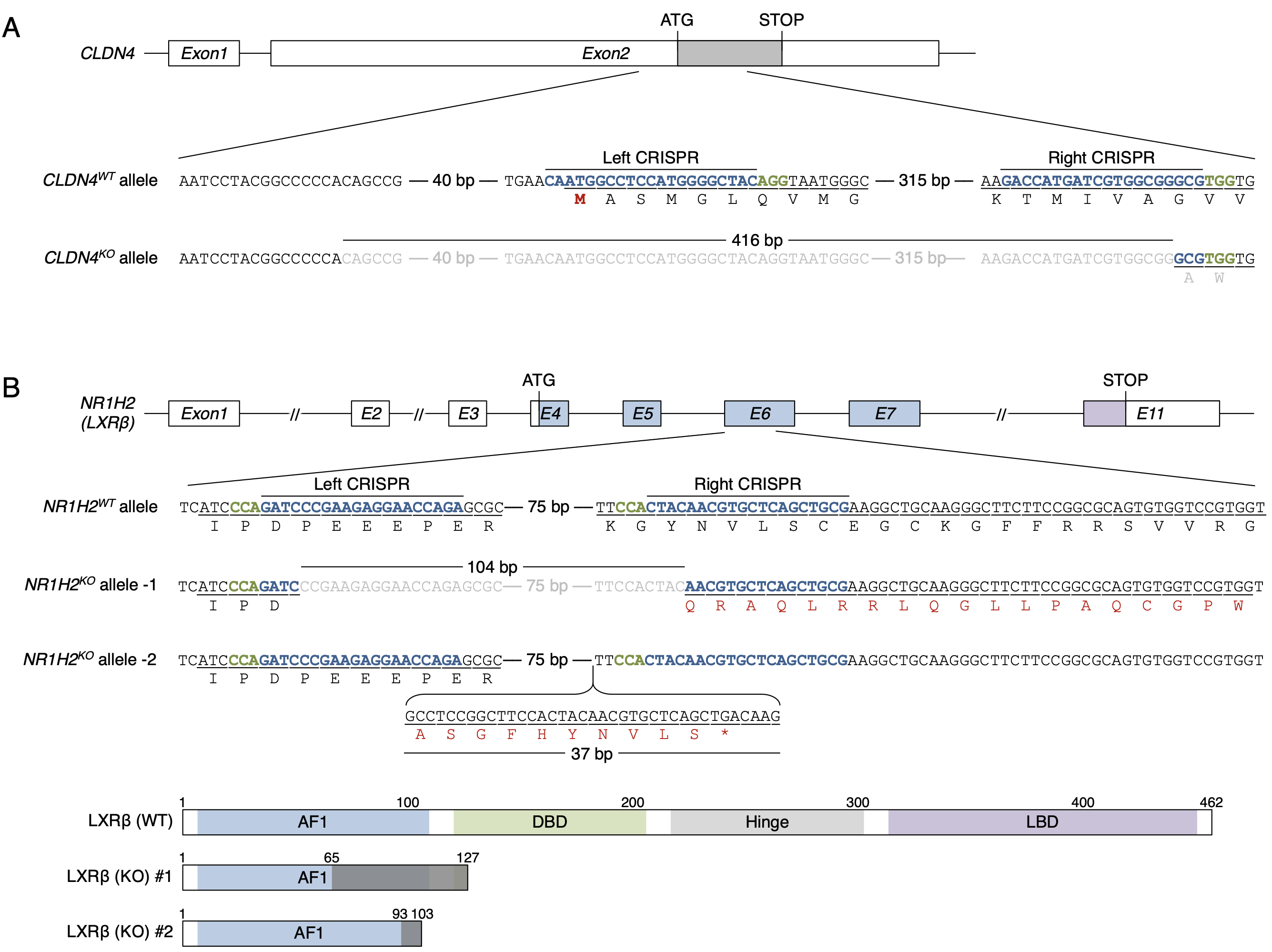
**

Fig. S2. **Knockout (KO) of the *CLDN4* and *LXRβ* genes in human breast cancer cell lines using the CRISPR/Cas9 method.** (A) KO of the *CLDN4* gene in T47D:*CLDN4^–/–^* and MCF-7:*CLDN4^–/–^* cells is confirmed by DNA sequencing. (B) KO of the *LXRβ* gene in T47D:*CLDN4^–/–^:LXRβ^–/–^* cells is verified by DNA sequencing.

**
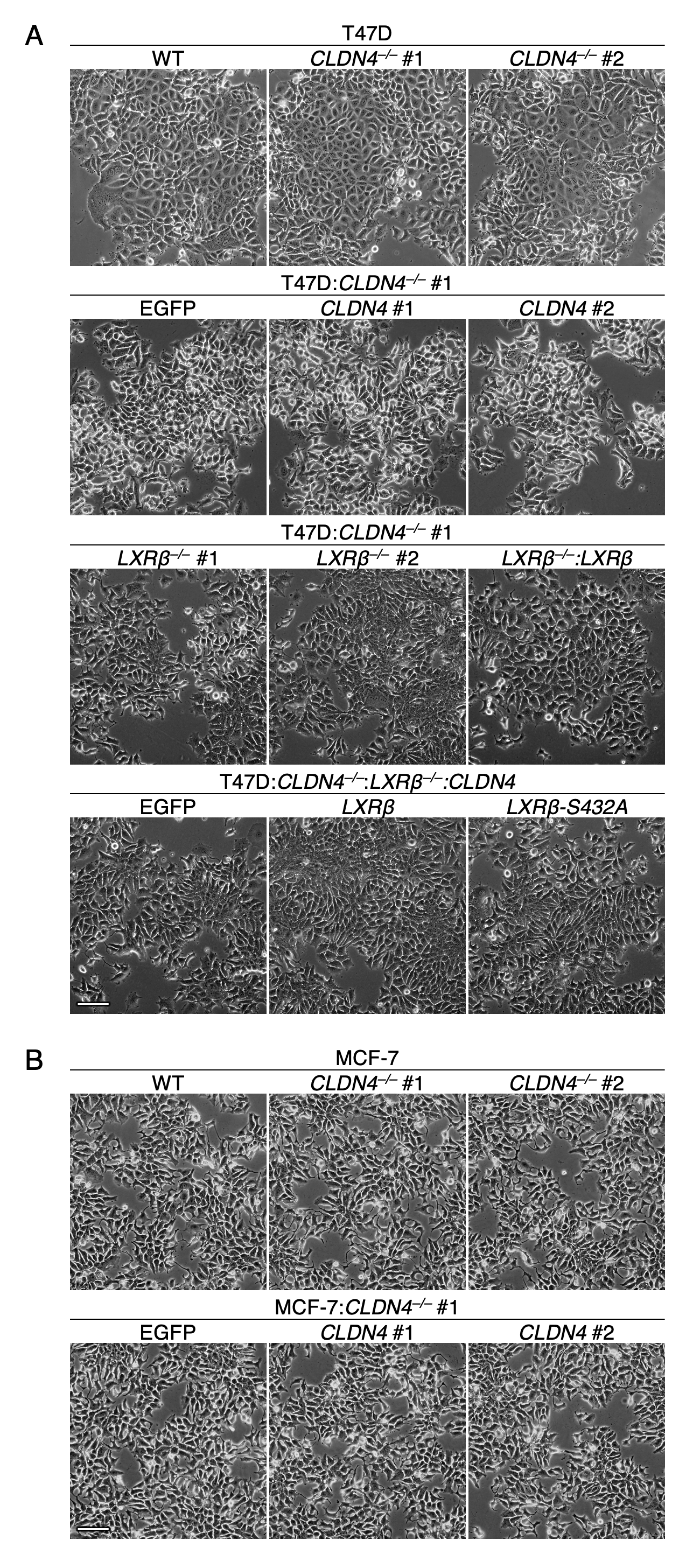
**

Fig. S3. **Phase-contrast images in the indicated cell lines.** (A and B) Representative images of each wild-type (WT) or transgenic T47D (A) and MCF-7 (B) cell line are shown. Scale bars, 100 μm.


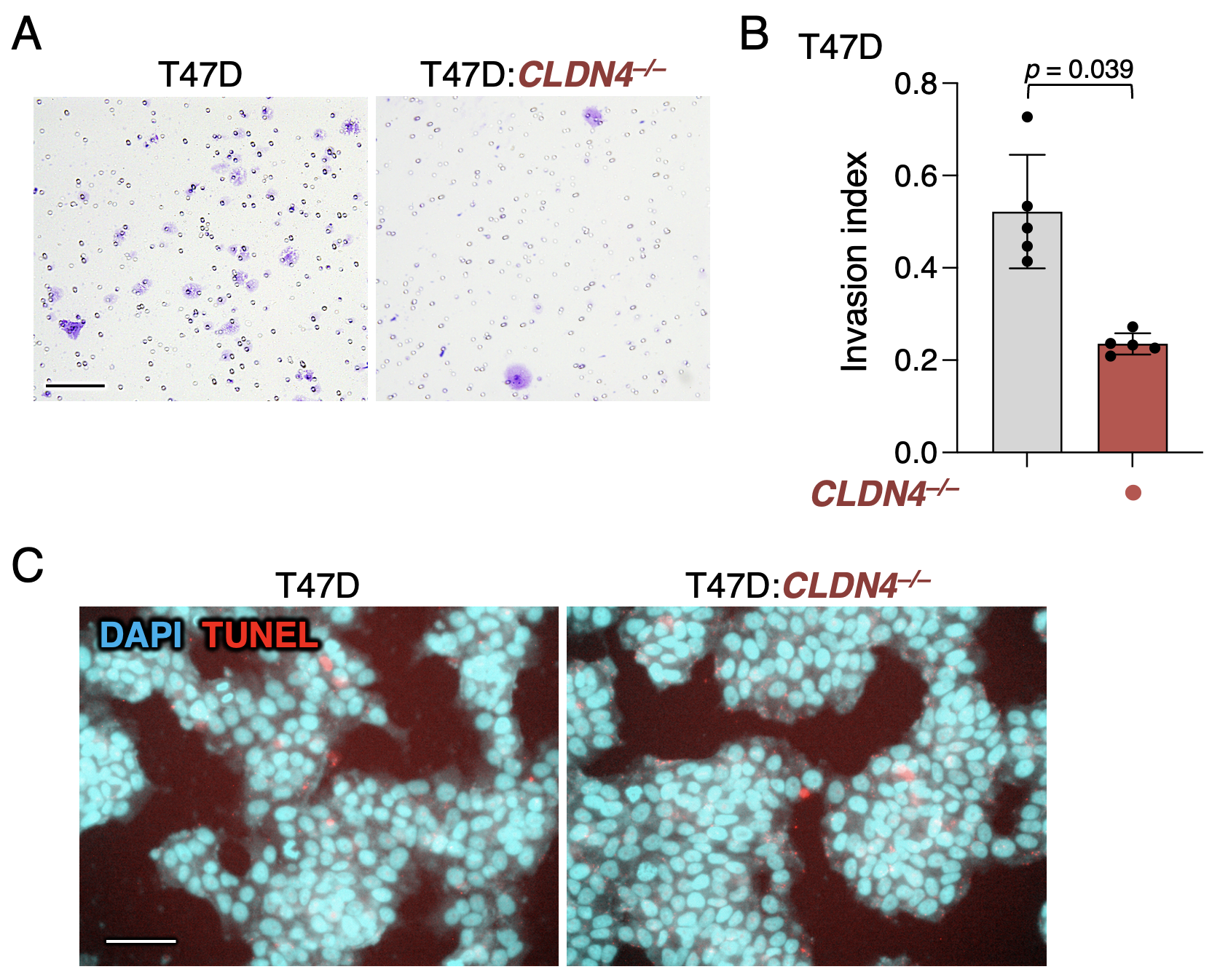


Fig. S4. **CLDN4 enhances cell invasion in the breast cancer cell line T47D.** (A and B) Representative and quantitative invasion assay for the indicated cells. The invasion index is plotted and shown in the histograms (mean ± SD; *n* = 5). (C) The absence of CLDN4 does not affect apoptosis in T47D cells. Cells are subjected to TUNEL assay together with DAPI staining. Scale bars, 100 μm.

**
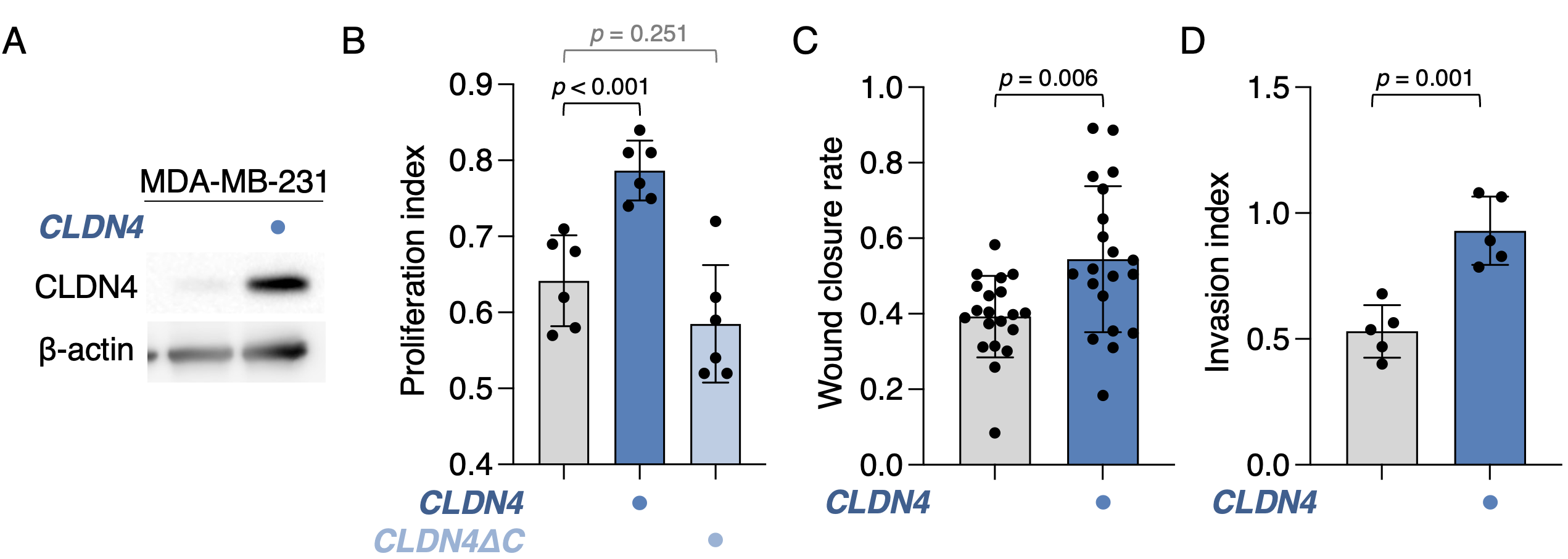
**

Fig. S5. **CLDN4 accelerates malignant activities of the breast cancer cell line MDA-MB-231.**(A) Western blot analysis indicating overexpression of CLDN4 protein in MDA-MB-231:*CLDN4* cells. (B) BrdU assay for the indicated cells. The BrdU/DAPI levels are plotted and shown in the histograms (mean ± SD; *n* = 6). (C) Wound healing assay of the indicated cells. The wound closure rates are plotted and shown in the histograms (mean ± SD; *n* = 20). (D) Invasion assay for the indicated cells. The invasion index is plotted and shown in the histograms (mean ± SD; *n* = 5).

**
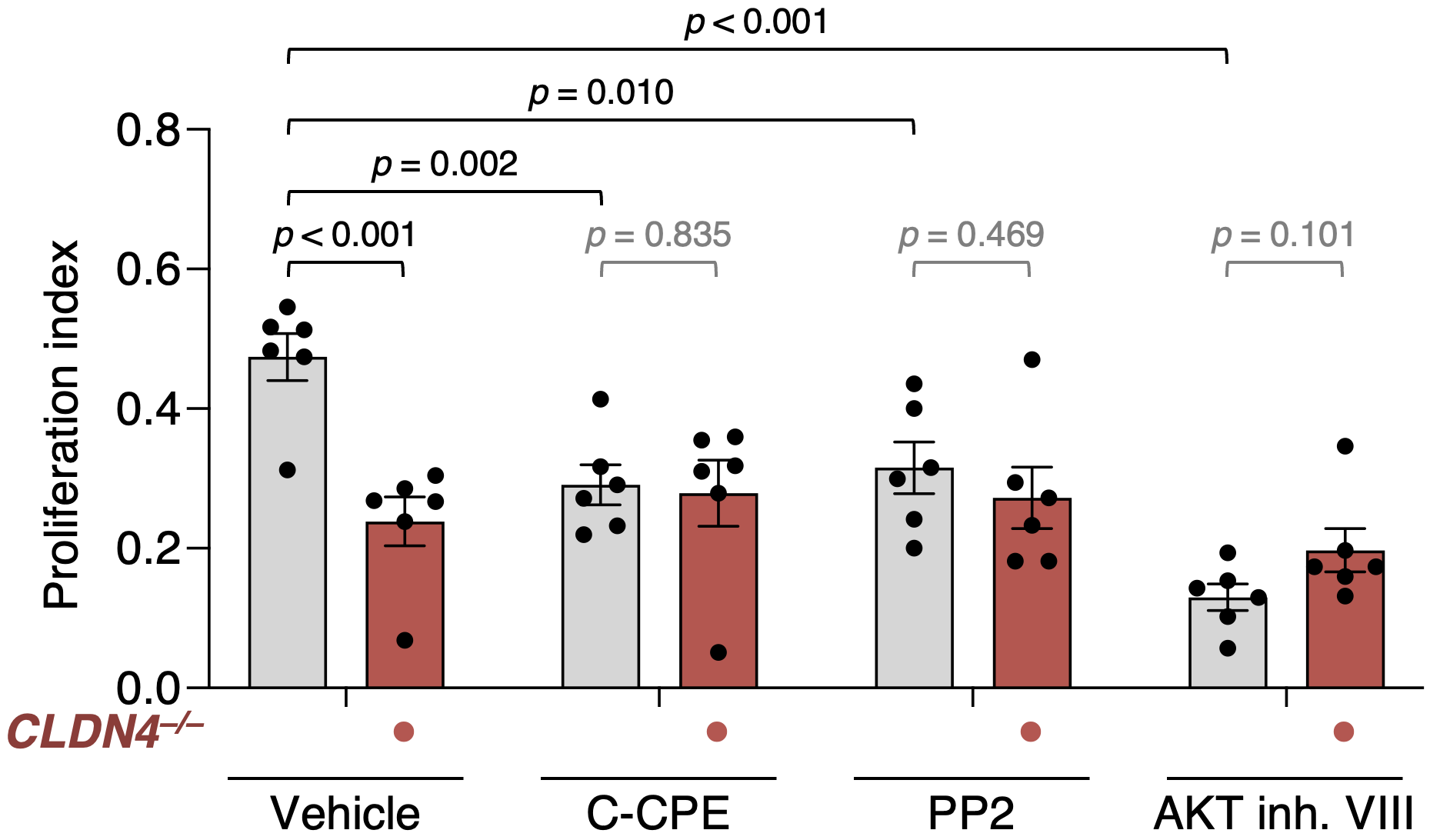
**

Fig. S6. **The C-terminal cytoplasmic domain of CLDN4, SFK, and AKT are involved in the CLDN4-accelerated breast cancer proliferation.** T47D and T47D:*CLDN4^–/–^* cells were grown for 24 h in the presence of vehicle, C-CPE (C-terminal half of *Clostridium Perfringens* enterotoxin; 1 μg/ml), the SFK inhibitor PP2 (10 μM), or the AKT inhibitor AKT inhibitor VIII (0.1 μM). The BrdU/DAPI levels are shown in histograms (mean ± SD; *n* = 6).

**
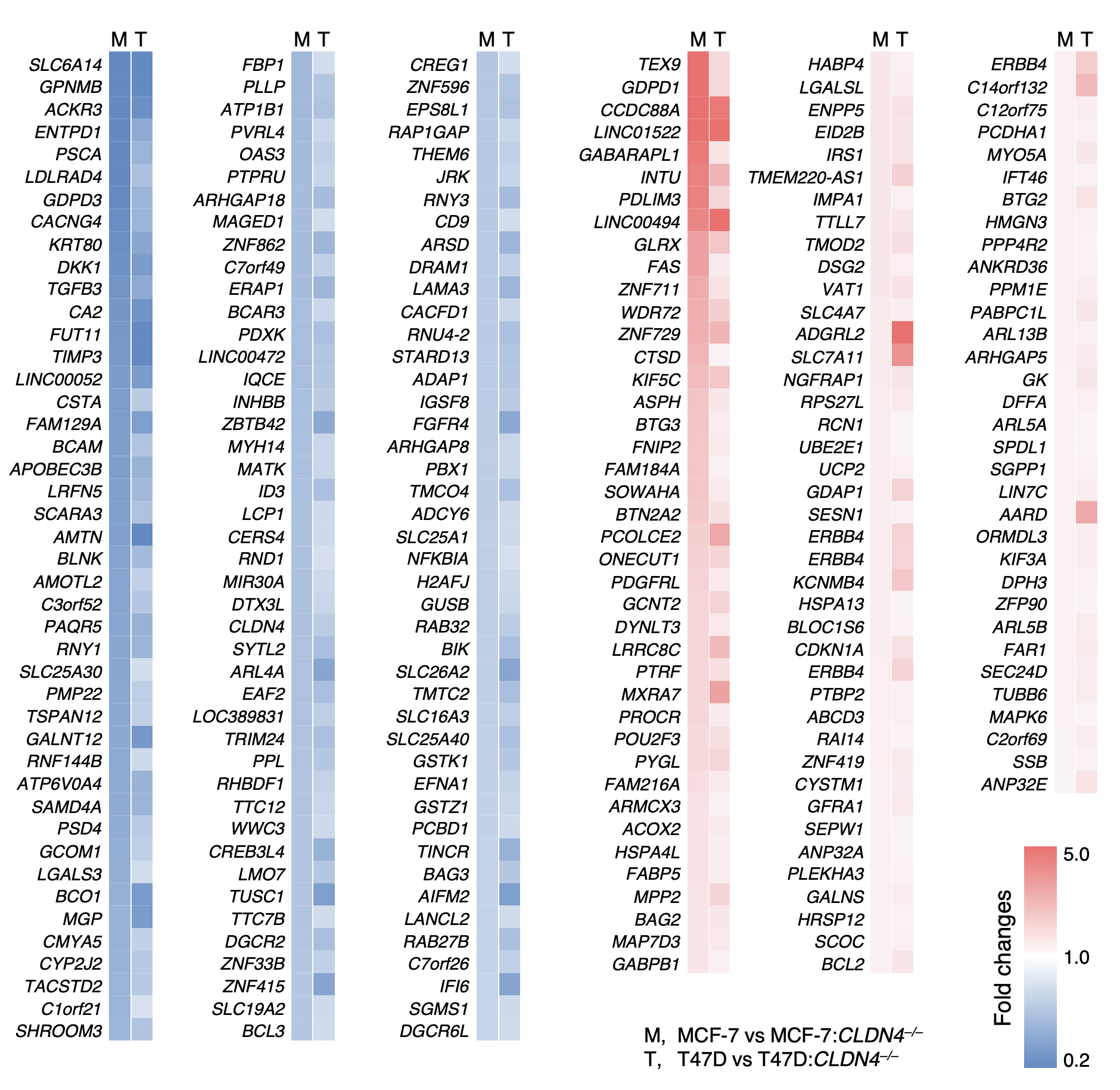
**

Fig. S7. **RNA sequence analysis showing that the CLDN4 signaling controls the expression of various genes in breast cancer cells.** Genes whose expression was significantly down- or up-regulated in both T47D:*CLDN4^–/–^* and MCF-7:*CLDN4^–/–^* cells compared with their parental cells (*p* < 0.05) are shown in the heatmap. Two batches of each cell line were subjected to RNA sequence analysis, and the average of fold changes of the revealed genes is indicated.

**
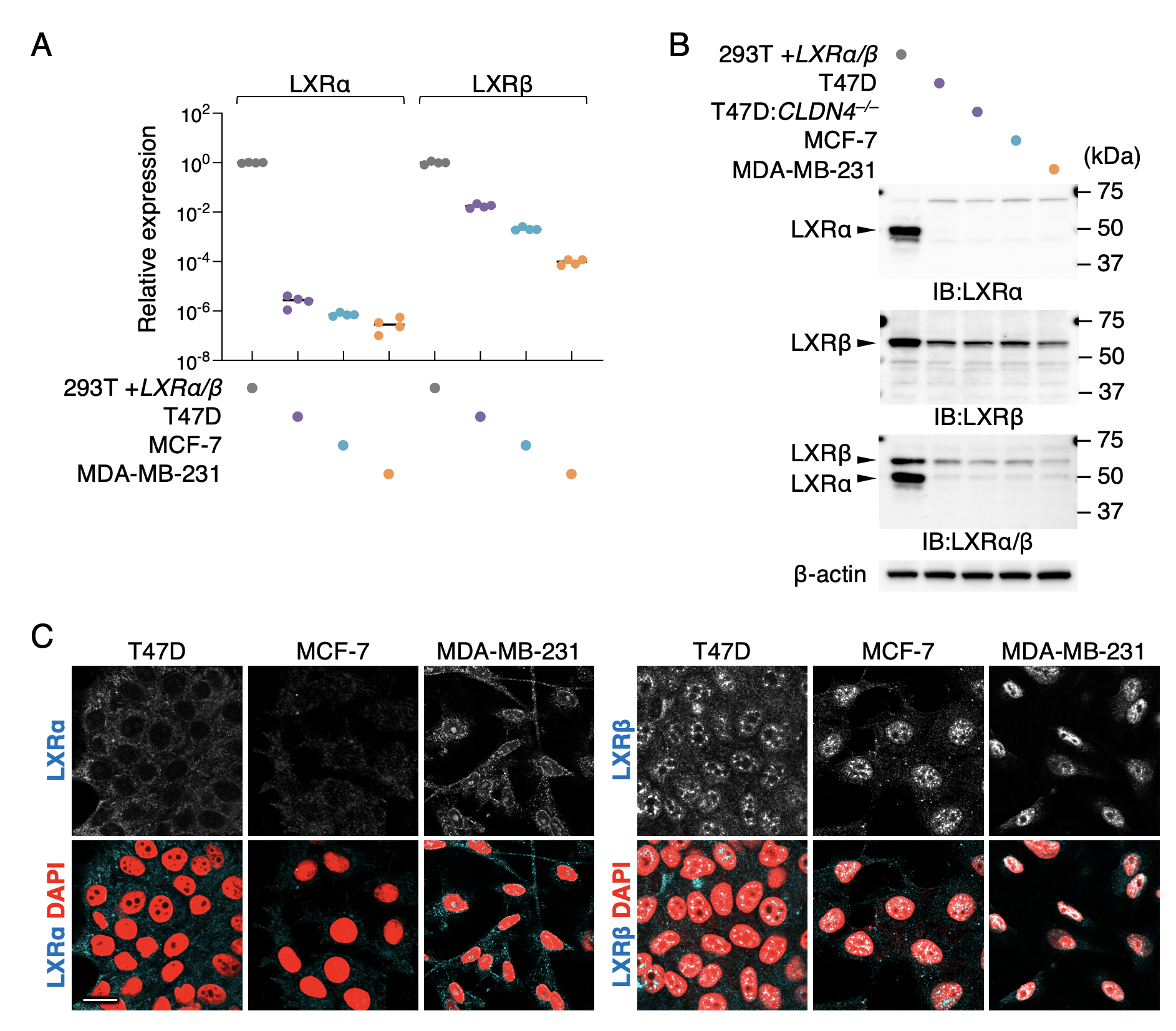
**

Fig. S8. **LXRβ is predominantly expressed in MCF-7, T47D, and MDA-MB-231 cells.** (A) RT-qPCR analysis for expression of *LXRα* and *LXRβ* mRNA. *LXRα*- or *LXRβ*-expressing HEK293T cells are used for positive controls, and the expression levels relative to *GAPDH* are shown as 1. The values are plotted, and the average is indicated as bars. (B) Western blot analysis for the indicated proteins in the revealed cells. A mixture of whole-cell extracts from HEK293T cells overexpressing LXRα and LXRβ is used as a positive control. (C) Confocal images of the indicated proteins in the revealed cell lines. Scale bar, 50 μm.


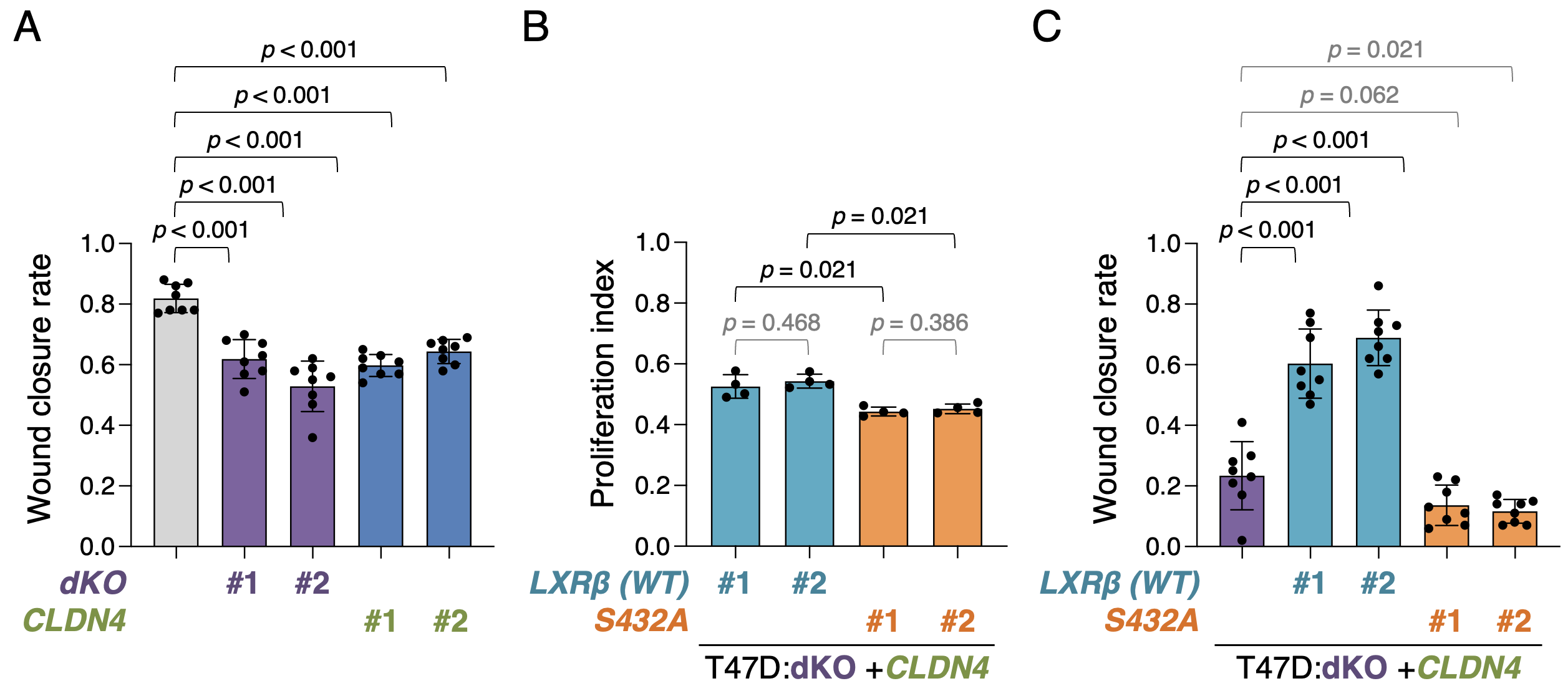


Fig. S9. **LXRβS432 is essential for the CLDN4-triggered breast cancer progression.** (A and C) Wound healing assay of the revealed T47D cells. The wound closure rates are plotted and shown in the histograms (mean ± SD; *n* = 8). (B) BrdU assay for the indicated T47D cells. The BrdU/DAPI levels are plotted and shown in the histograms (mean ± SD; *n* = 4). dKO, *CLDN4^–/–^:LXRβ ^–/–^*.


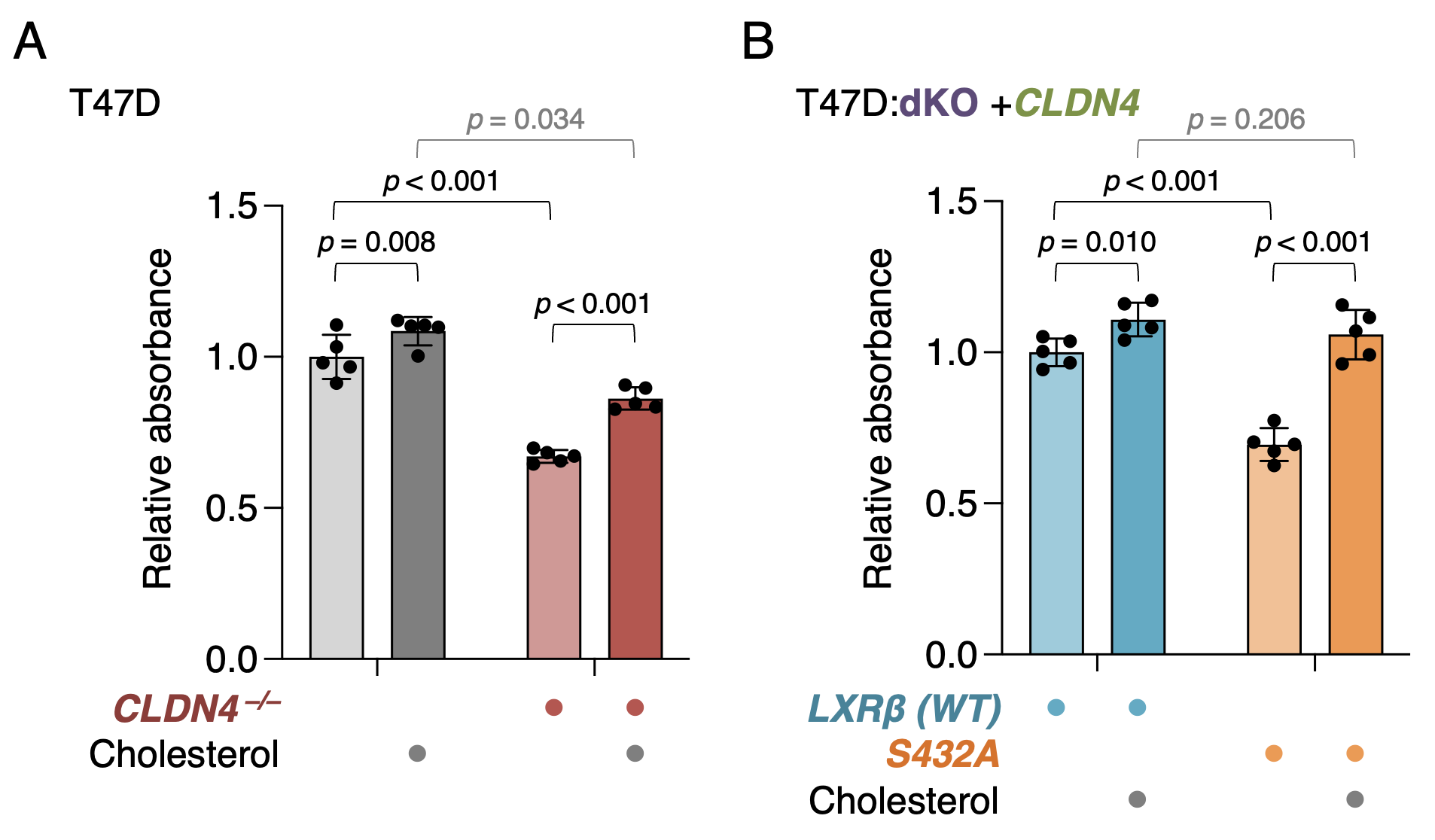


Fig. S10. **Treatment of T47D:*CLDN4^–/–^* (A) and T47D:dKO:*CLDN4:LXRβS432A* (B) cells with cholesterol recovers cell viability.** The indicated T47D cells were grown for 24 h in the presence or absence of 1 mg/ml cholesterol. The relative cell numbers are plotted and shown in histograms (mean ± SD; *n* = 5). dKO, *CLDN4^–/–^:LXRβ ^–/–^*.


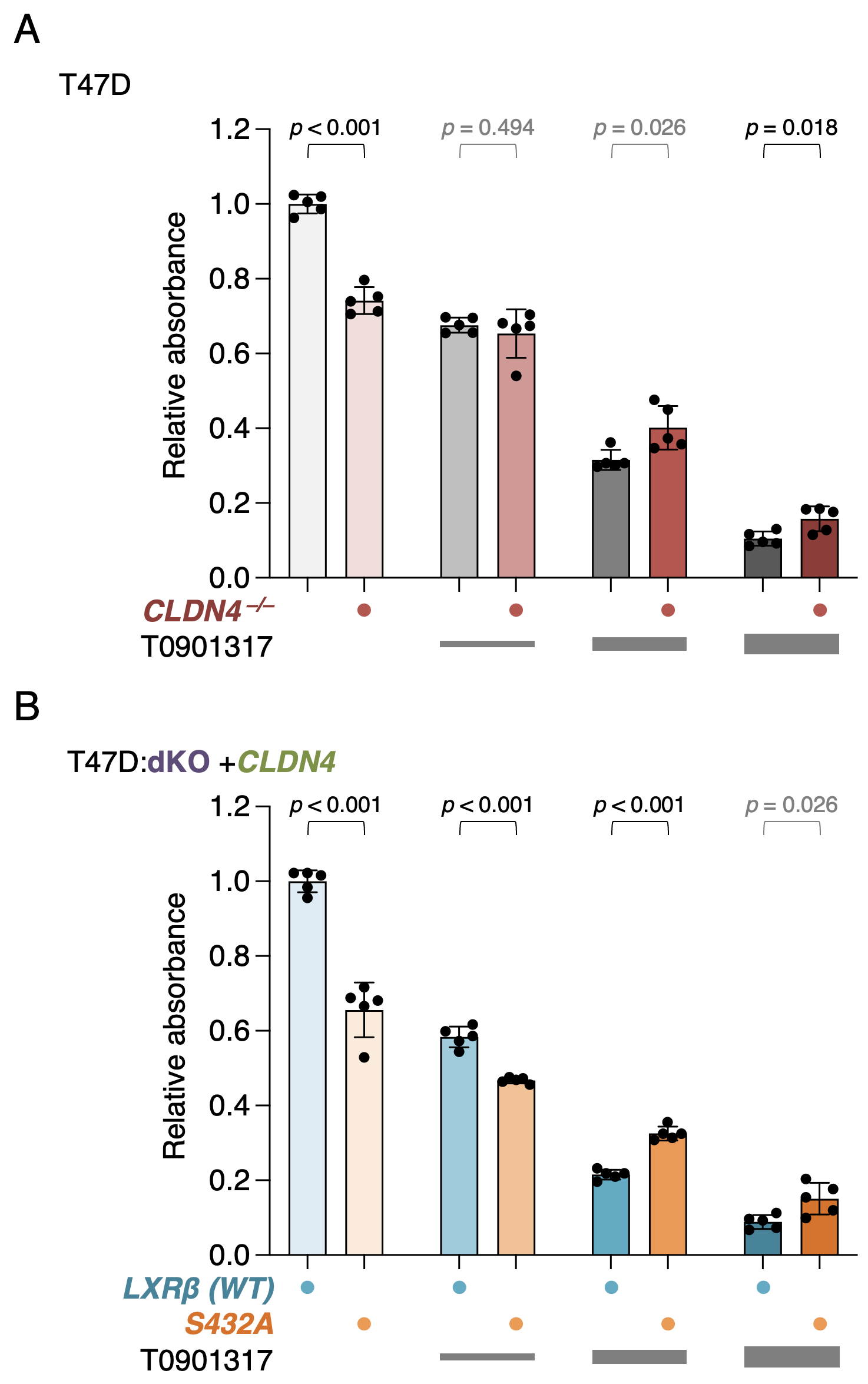


Fig. S11. **Effect of a synthetic LXR ligand T0901317 on cell viability of T47D, T47D:*CLDN4^–/–^*, T47D:dKO:*CLDN4:LXRβ*, and T47D:dKO:*CLDN4:LXRβS432A* cells.** The indicated T47D cells were grown for 24 h in the presence or absence of 1, 5, and 25 μM T0901317. The relative cell numbers are plotted and shown in histograms (mean ± SD; *n* = 5). dKO, *CLDN4^–/–^:LXRβ ^–/–^*.

**
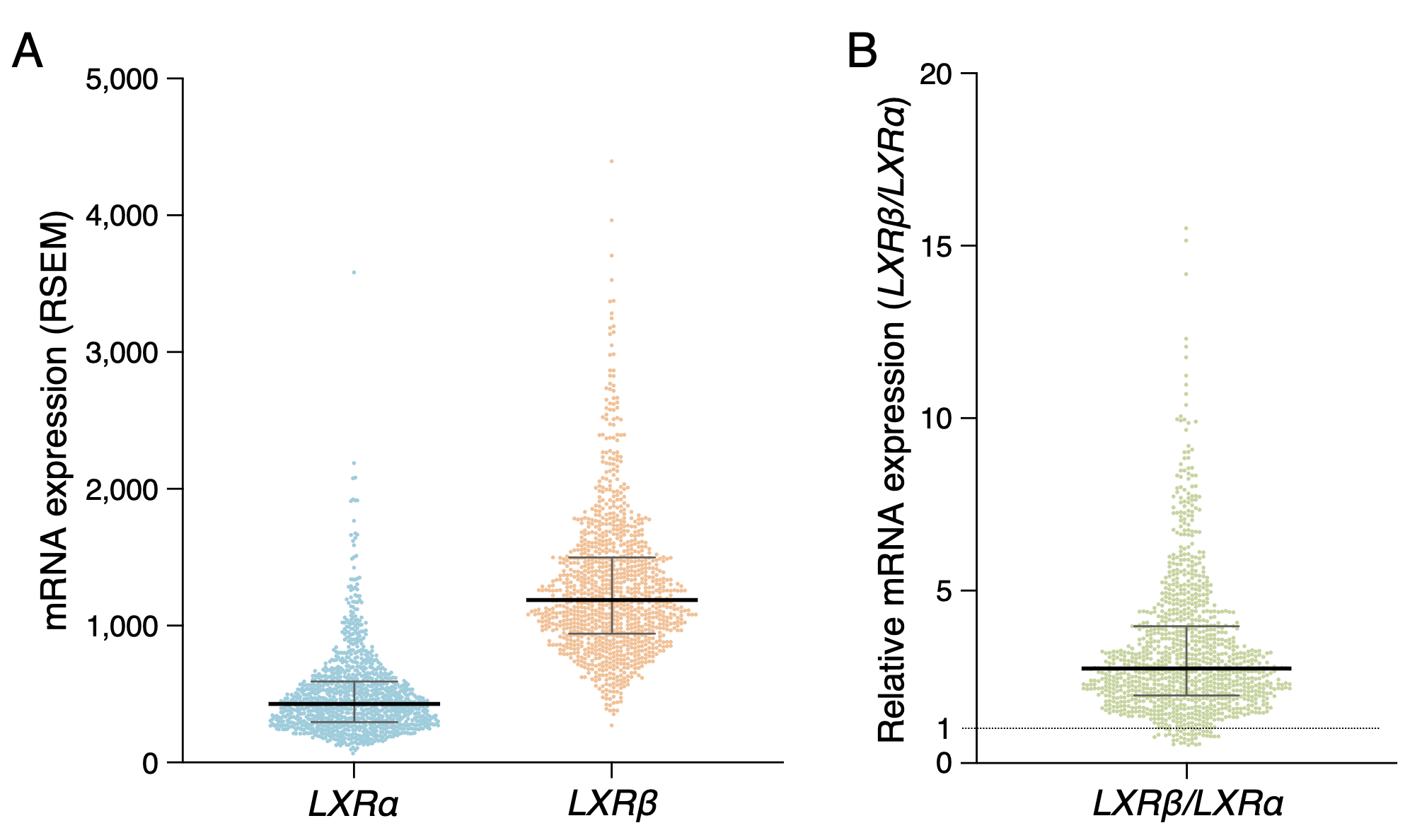
**

Fig. S12. ***LXRβ* mRNA is mainly expressed in breast cancer subjects.** (A and B) Expression of *LXRα* and *LXRβ* transcripts in 1,100 breast cancer tissues using The Cancer Genome Atlas (TCGA) database. RSEM values of *LXRα* and *LXRβ* (A) and mRNA expression of *LXRβ* relative to *LXRα* (B) are shown as median and interquartile range.

**
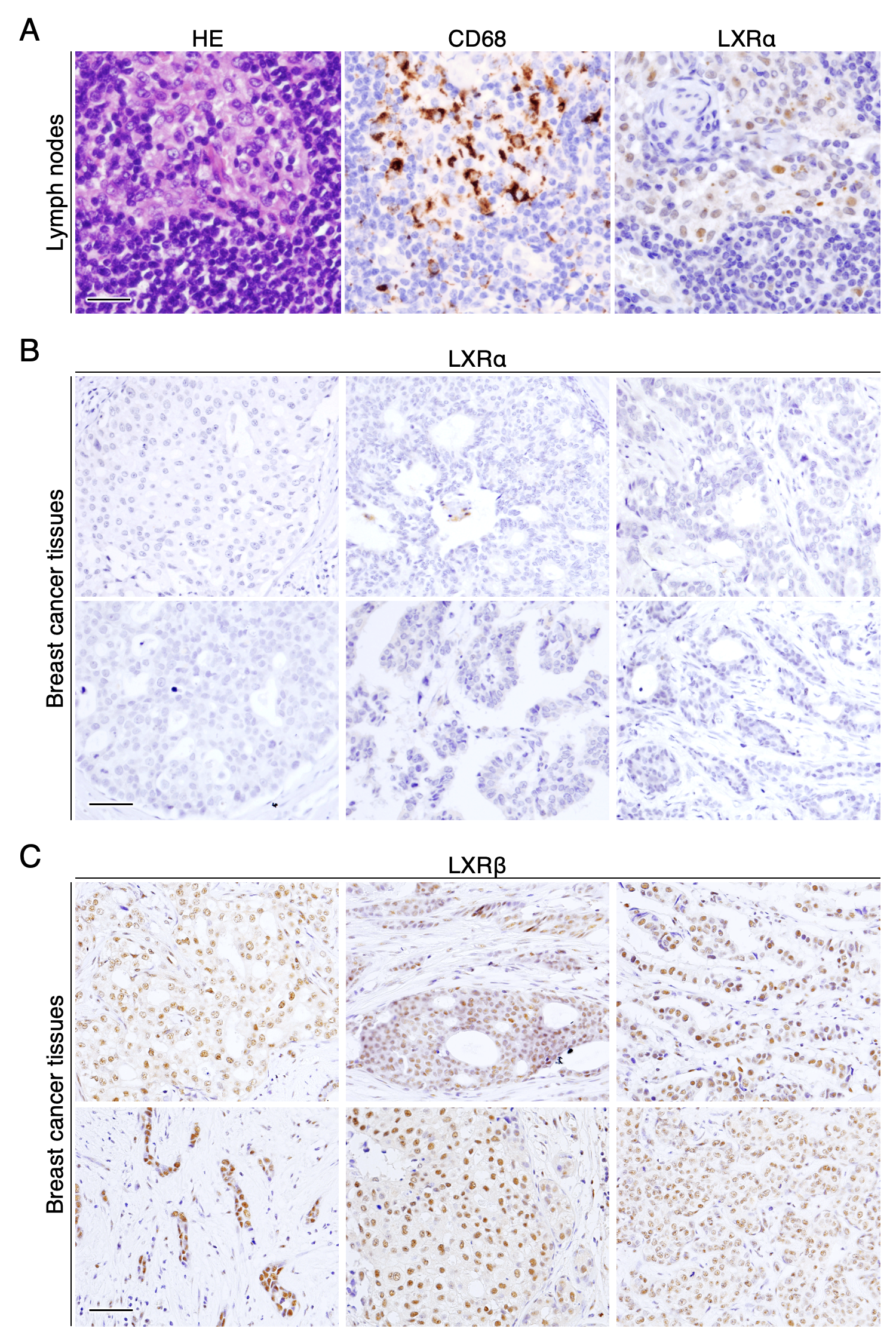
**

Fig. S13. **LXRβ but not LXRα is observed in breast cancer tissues.** (A) Representative immunohistochemical images for CD68 and LXRα in a lymph node of breast cancer patients. HE, hematoxylin-eosin. Scale bar, 50 µm. (B and C) Representative immunohistochemical images for LXRα (B) and LXRβ (C) in breast cancer tissues. Scale bars, 100 μm.**
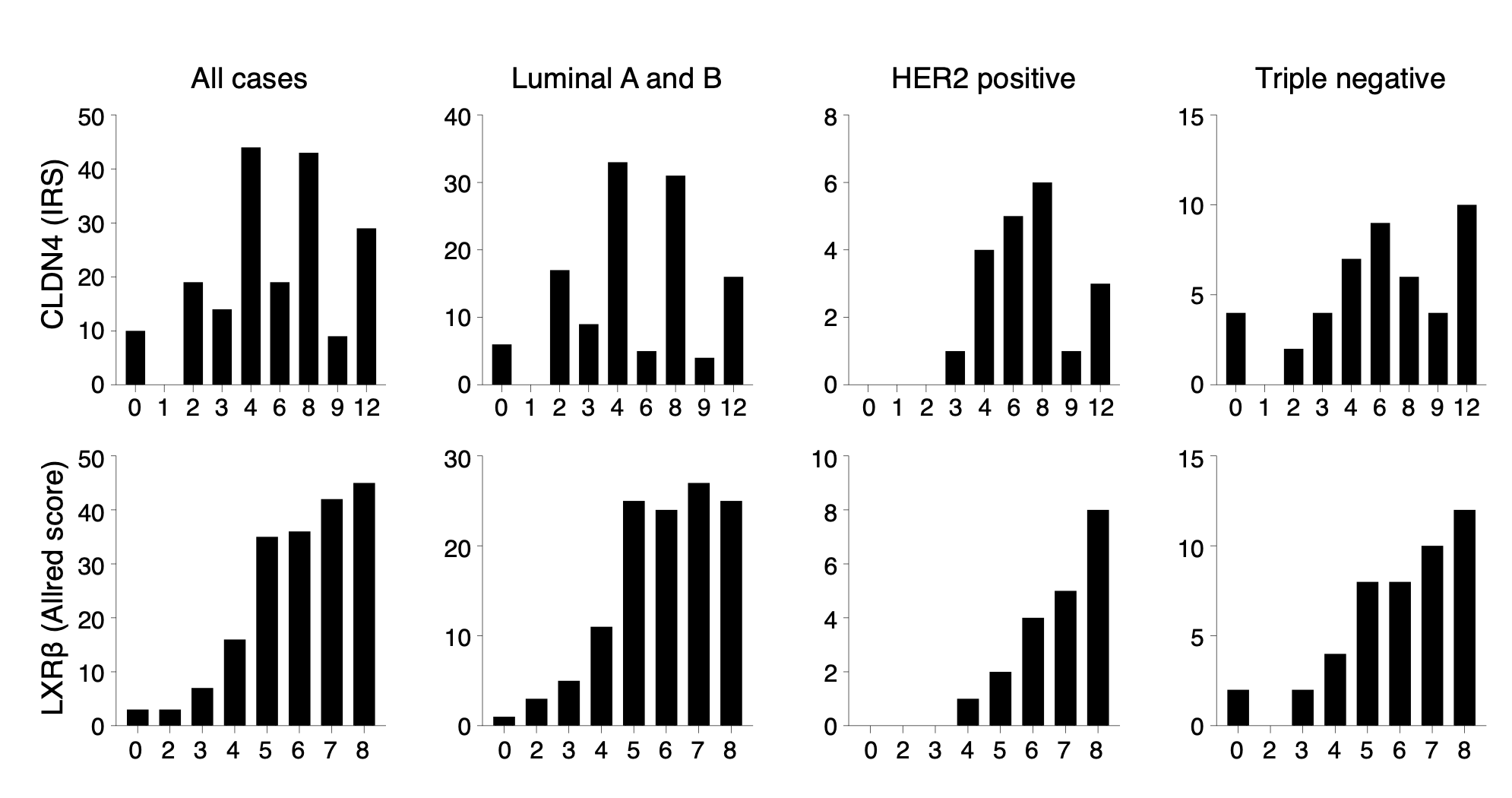
**

Fig. S14. **Semi-quantification of the CLDN4 and LXRβ expression in the indicated breast cancer subjects.** IRS, immunoreactive score.

**
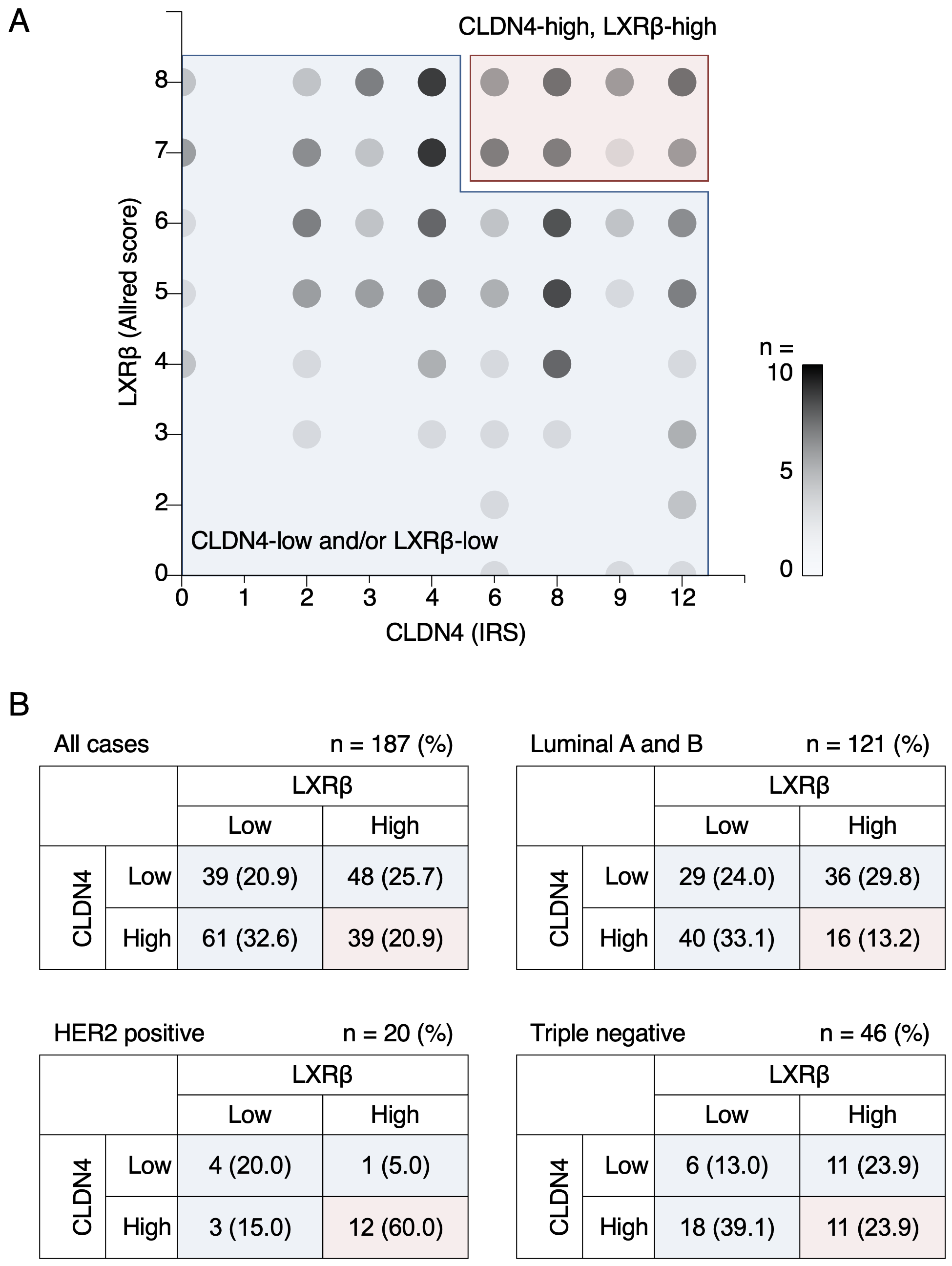
**

Fig. S15. **Classification of breast cancer subjects by expression levels of CLDN4 and LXRβ.** (A) The CLDN4 and LXRβ scores are shown as a bubble dot. IRS, immunoreactive score. (B) The number of "CLDN4-high/LXRβ-high" and "CLDN4-low and/or LXRβ-low" groups in the revealed breast cancer cases are indicated.

**
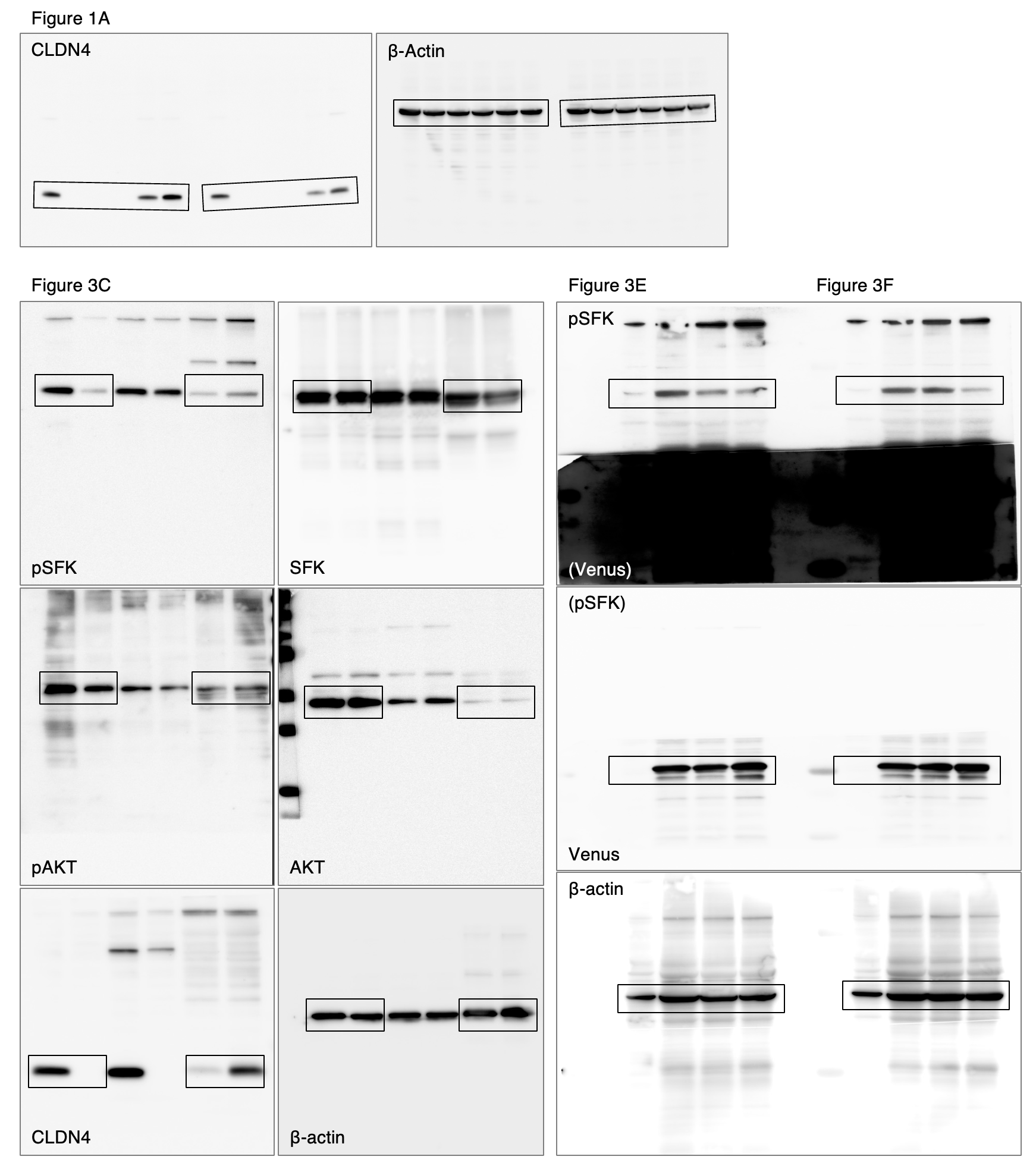
**(Fig. S16; continuing)

**
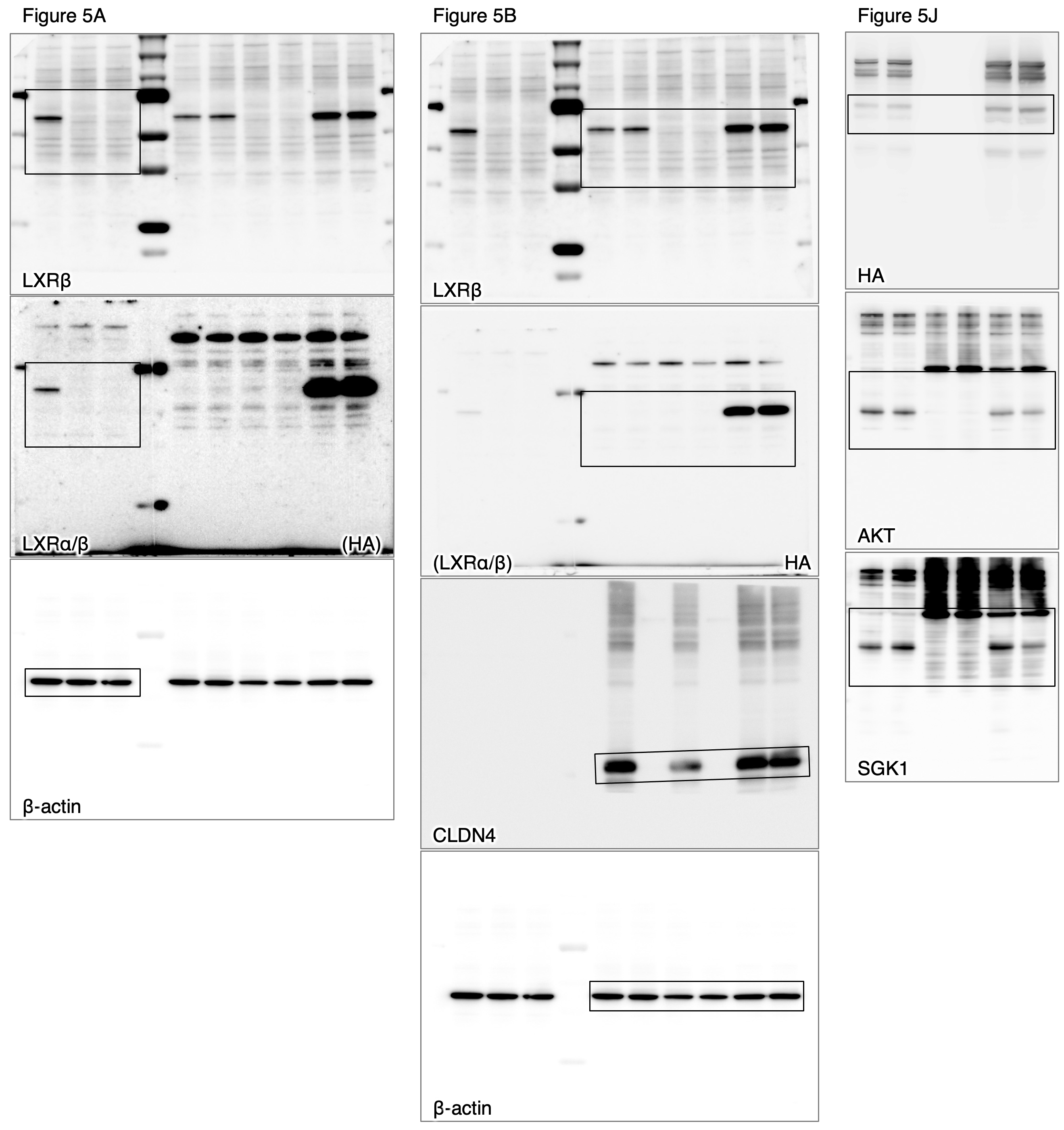
**(Fig. S16; continuing)

**
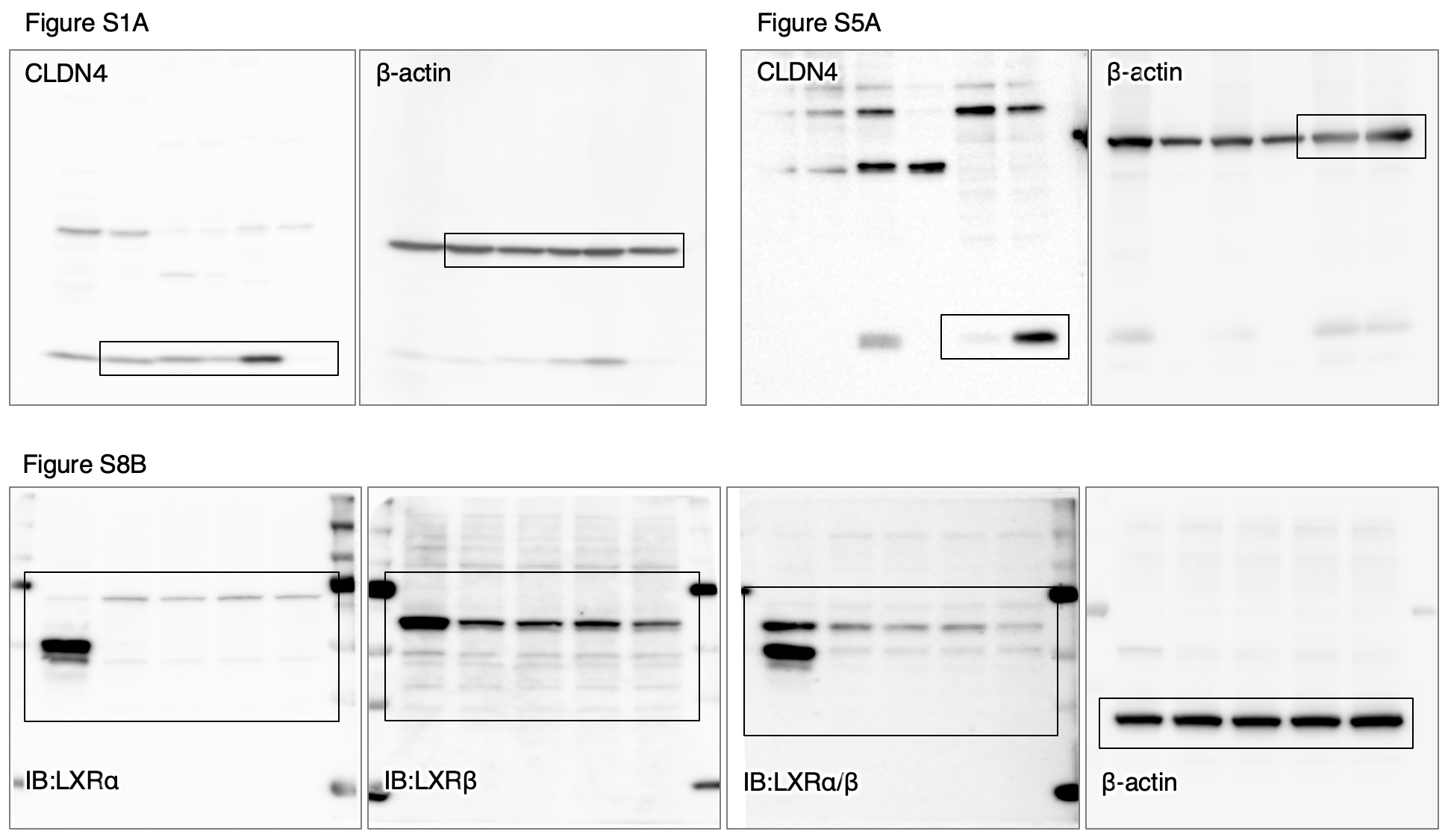
**

Fig. S16. **Uncropped images for the indicated Western blot.**

Table S1. **Clinicopathological characteristics of the patients with breast cancer.**

HER2, human epithelial growth factor receptor type 2; TNBC, triple-negative breast cancer; DCIS, ductal carcinoma in situ; IDC, invasive ductal carcinoma; ILC, invasive lobular carcinoma

Table S2. **Antibodies**

IF, immunofluorescence; IHC, immunohistochemistry; WB, western blotting; IP, immunoprecipitation;
m, monoclonal; p, polyclonal

Table S3. **Primers**


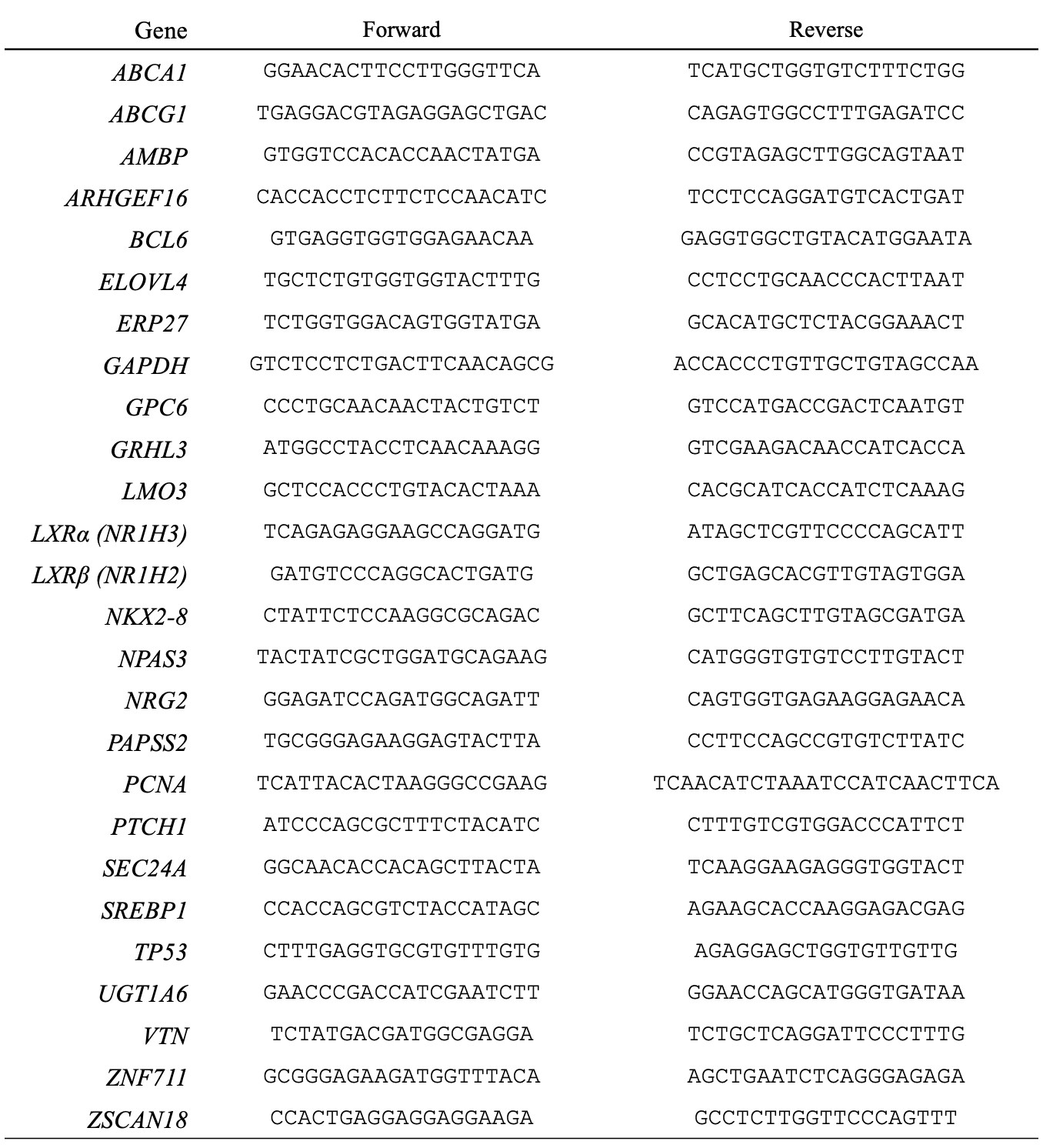
Table S4. **Immunoreactive Score (IRS)**
